# Cancer incidence in patients with ulcerative colitis naïve to or treated with thiopurine and targeted therapies—a cohort study 2007 to 2022 with comparison to the general population

**DOI:** 10.1093/ecco-jcc/jjaf091

**Published:** 2025-06-02

**Authors:** Åsa H Everhov, Johan Askling, Jonas Söderling, Jonas Halfvarson, Julia Eriksson, Karin E Smedby, Jonas F Ludvigsson, Henrik Toft Sørensen, Ola Olén

**Affiliations:** Department of Clinical Science and Education, Södersjukhuset, Karolinska Institutet, Stockholm, Sweden; Clinical Epidemiology Division, Department of Medicine Solna, Karolinska Institutet, Stockholm, Sweden; Department Medical Epidemiology and Biostatistics, Karolinska Institutet, Stockholm, Sweden; Clinical Epidemiology Division, Department of Medicine Solna, Karolinska Institutet, Stockholm, Sweden; Clinical Epidemiology Division, Department of Medicine Solna, Karolinska Institutet, Stockholm, Sweden; Department of Gastroenterology, Faculty of Medicine and Health, Örebro University, Örebro, Sweden; Centre for Pharmacoepidemiology, Department of Medicine Solna, Karolinska Institutet, Stockholm, Sweden; Clinical Epidemiology Division, Department of Medicine Solna, Karolinska Institutet, Stockholm, Sweden; Department of Hematology, Theme Cancer, Karolinska University Hospital, Stockholm, Sweden; Department Medical Epidemiology and Biostatistics, Karolinska Institutet, Stockholm, Sweden; Department of Pediatrics, Örebro University Hospital, Örebro University, Örebro, Sweden; Department of Clinical Epidemiology, Aarhus University Hospital and Aarhus University, Aarhus, Denmark; Clinical Epidemiology Division, Department of Medicine Solna, Karolinska Institutet, Stockholm, Sweden; Department of Pediatric Gastroenterology and Nutrition, Sachs’ Children and Youth Hospital, Stockholm, Sweden

**Keywords:** inflammatory bowel disease, ulcerative colitis, thiopurine, tumor necrosis factor inhibitor, vedolizumab, ustekinumab, tofacitinib, cancer, incidence, population-based

## Abstract

**Background:**

Cancer incidence data including absolute risk differences are needed for clinical risk communication to patients receiving modern-day treatments for ulcerative colitis (UC).

**Methods:**

We linked nationwide Swedish health registers and assessed incident cancers in patients with UC in 2007-2022. We computed age-stratified incidence rates (IRs), IR differences, and hazard ratios (HRs) in a naïve cohort with no immunomodulatory treatment, and in cohorts treated with thiopurine or targeted therapies. General population comparator subjects were matched (by age, sex, calendar year, and area of residence) to each treatment cohort. We used a once-exposed—always-exposed design.

**Results:**

We identified 63 925 patients with UC in partly overlapping cohorts and 593 072 comparators with a total follow-up time of 5 800 089 years (median 8.1 years). The IRs were elevated compared to the general population in naïve patients: 2.7 extra cancer cases per 1000 person-years (HR: 1.12, 95% CI, 1.09-1.16), in thiopurine-treated patients: 3.4 extra cases (HR: 1.48;1.37-1.61), tumor necrosis factor inhibitor (TNFi)-treated: 2.7 extra cases (HR: 1.41;1.24-1.62), Thiopurine + TNFi-treated: 2.42 extra cases (HR: 1.44;1.19-1.75), vedolizumab-treated: 2.88 extra cases (HR: 1.27;0.90-1.79). The IR differences were not significantly increased in patients treated with ustekinumab 0.57 (HR: 0.87;0,39-1.93) and tofacitinib −0.69 (HR: 0.84;0.25-2.77). Across all treatment groups, the IR differences compared to the general population were highest in patients ≥ 60 years. The differences were driven by colorectal cancer, hepatobiliary cancer, lymphoma, and basal cell skin carcinoma.

**Conclusions:**

Elevated cancer incidence was observed in patients with UC amounting to around 3 extra cases of cancer per 1000 years. Cancer risks varied more among groups defined by age than by treatment.

## 1. Introduction

Ulcerative colitis (UC) is an inflammatory bowel disease (IBD) characterized by chronic inflammation of the colon and rectum, due to an abnormal immune response to environmental factors among genetically susceptible individuals.^1^ Medical therapies such as thiopurines, tumor necrosis factor inhibitors (TNFi), and other targeted therapies (TTs), which decrease inflammation, are used as medical treatment of UC and should preferably be administered early in the disease course to avoid irreversible tissue damage.^2^

Cancers in patients with UC can arise through several mechanisms: they can be sporadic and unrelated to the disease, they can share risk factors with UC, be caused by the disease, for example, through chronic inflammation, or be causally linked with the treatment itself.^3,4^ At the same time, improved disease control via effective treatment might prevent or reduce a disease-mediated increased risk. Studies have demonstrated that, across all treatment types, certain gastrointestinal cancers, such as colorectal cancer (CRC)^5–8^ and hepatobiliary cancer^9,10^ are more common in patients with UC than in the population. Patients with UC are also at elevated risk of extraintestinal cancers.^11^

Investigations of the association between cancer and UC medication have focused primarily on thiopurines.^12–18^ In meta-analyses of studies comparing patients with IBD with various treatment exposures, thiopurines have been associated with diminished risk of CRC,^13,14,19^ but elevated risks of skin cancer^17,18^ and lymphoma.^16,20^ To date, meta-analyses based on non-experimental,^12,21–26^ and placebo-controlled trials,^27^ have not linked TNFi use in IBD to increased risk of overall cancer risk,^21,22,27^ lymphoma,^23^ melanoma,^24,25^ or cervical cancer,^26^ except for one study, indicating a slightly greater risk of lymphoma in TNFi users than non-users.^20^ Newer TTs have mainly been evaluated within randomized controlled studies with short follow-up time^28,29^ (Table S1).

For clinical risk communication and decision-making, HR is a difficult metric because it conveys the relative change without specifying the absolute risk. In this study, the aim was to provide an overview of the absolute and excess occurrence of cancer in patients with UC with or without exposure to specific UC drugs vs the cancer incidence in the general population with the same age/sex composition.

## 2. Methods

### 2.1. Study design

Nationwide cohort study based on national health registers with prospectively captured data from routine medical practice.

### 2.2. Setting

Health care in Sweden is tax-funded. All citizens have equal access to care. The unique personal identity number assigned to all residents allows for the linkage of data in nationwide registers containing information on vital status, emigration, morbidity, mortality, and histopathology, with complete follow-up.^30^ In Sweden, treatment with thiopurines, primarily azathioprine, was initiated during the early/mid 1980s. Infliximab, the first TNFi, was approved for the treatment of UC in 2006, adalimumab in 2012, and golimumab in 2013. Vedolizumab (the first Integrin inhibitor) was approved in 2014, tofacitinib (the first Janus kinase inhibitor) in 2018, and ustekinumab (an Interleukin inhibitor) in 2019 (Figure S1).

### 2.3. Data sources

Baseline and follow-up data, including patient demographics, disease characteristics, treatments, and outcomes were obtained from diagnostic listings in the National Quality Register SWIBREG,^31^ the Swedish National Patient Register,^32^ the Prescribed Drug Register,^33,34^ the Swedish Cancer Register,^35^ and the Total Population Register (Table S2).^36^

### 2.4. Participants

We identified all Swedish patients with UC. Patients with incident UC were included from January 1, 2007 to December 31, 2021 if they had ≥2 first-ever listings of diagnostic codes of UC in non-primary outpatient clinic or inpatient care. Patients with prevalent UC were identified as those with ≥2 listings of UC before July 1, 2008 and—in case of different IBD subtype diagnoses—the last 2 diagnostic listings (before July 1, 2008) indicating UC.^37,38^ Each patient was matched by age, sex, and place of residence with up to 10 general population comparator subjects who were free of IBD on the date of the first UC diagnosis of the matched case (diagnostic codes provided in Table S3). Exclusion criteria at baseline for all participants were: (1) absolute or relative contraindication to thiopurine and/or TNFi (ie, human immunodeficiency virus, HIV, chronic hepatitis or other advanced liver disease, solid organ or bone marrow transplantation, or advanced kidney disease), or (2) previous use of immunomodulators (ie, azathioprine, mercaptopurine, or methotrexate), TNFi, or other TTs (ie, vedolizumab, ustekinumab, or tofacitinib) before the UC diagnosis/match date (Table S4). Participants with any previous invasive cancer were excluded from the analyses of any cancer. For cancer subtype-specific outcomes, participants were only excluded if they had a history of the same outcome/cancer type as that under study.

### 2.5. Exposure

The exposures of interest were: *thiopurine*, *TNFi* (infliximab, adalumumab, or golimumab), *thiopurine + TNFi*, that is, combined treatment with thiopurine and TNFi, *vedolizumab*, *ustekinumab*, and *tofaniticib* (Table S5). To avoid detection and surveillance bias around UC diagnosis and follow-up, we employed a 1-year latency period between the exposure (date of UC diagnosis/initiation of the treatment under study) and outcome assessment (cancer diagnosis).

The outcomes were assessed in 7 treatment cohorts. Patients were considered exposed to a medication from 1 year after its initiation until the end of follow-up. For all participants, follow-up ended with an outcome event, death, emigration, or December 31, 2022 whichever occurred first. The start and end of follow-up in the treatment cohorts were: *naïve*, follow-up started 1 year after UC diagnosis in incident patients and from one year after July 1, 2008 in patients with prevalent UC, and, in addition to the end of follow-up, as described above, naïve patients were censored 1 year after the start of treatment with thiopurine, TNFi, vedolizumab, ustekinumab, or tofacitinib. For the drug exposures thiopurine, TNFi, vedolizumab, ustekinumab, and tofaniticib, patients were followed from 1 year after the first prescription redemption/treatment registration to the end of follow-up. For *thiopurine+TNFi* patients were considered exposed from 1 year after the first combined treatment episode (defined as at least 3 months’ overlapping exposure) to the end of follow-up. Patients could thus contribute person-time to multiple treatment groups and one cancer event could be assigned to multiple treatment groups. All statistical comparisons were performed vs matched general population comparators.

### 2.6. Outcome

Incident cancers were identified in the Cancer Register through *International Classification of Diseases,* (ICD) codes and the histopathological 3-digit code (C24), used since 1958. Diagnostic coding of the different cancer types is listed in Table S6.

We assessed incident cancers according to organ site, reported as:

- *Cancers associated with UC*: CRC, small bowel, pancreatic, and hepatobiliary cancer.
- *Cancers with known or suspected association with thiopurine or TNFi*: malignant melanoma, basal cell skin carcinoma, squamous cell carcinoma of the skin, lymphoma, other hematological malignancy, cervical cancer, and urinary tract cancer.^11^
- *Other common cancer forms*: breast cancer, prostate cancer, lung cancer, uterine cancer, and brain or spinal cord cancers.

### 2.7. Covariates

Diagnostic listings during the 5 years preceding the UC match date were used to characterize all study participants in terms of comorbid conditions (Table S7) and medication use (Table S8) occurring/dispensed before the matching date.^34^ Patients with UC were further characterized by surgery (colectomy or other bowel surgery, Table S9),^39^ and the presence of primary sclerosing cholangitis (PSC).

### 2.8. Statistical methods

Baseline characteristics are presented as number and proportion (%) for categorical variables, or as mean and SD or median and IQR for continuous variables. The cumulative incidences and incidence rates (IRs) (number of events/1000 person-years) of any cancer and cancer type are presented for each cohort, as well as standardized to the age- and sex-distribution of the Swedish population in 2023. The cumulative incidence of any cancer is presented stratified by age at the start of follow-up (<18, 18- <40y, 40- <60, ≥60 years). Differences between patient cohorts and matched population comparators are presented as IR differences and adjusted (age, sex, region, calendar year) hazard ratios (HRs) from Cox regression models with 95% CIs overall and stratified by age at exposure start. The proportional hazard assumption was tested by the inclusion of an interaction term of exposure and the time scale (follow-up time). Only patients with at least one available population comparator subject were included for each outcome.

All statistical tests were 2-sided, and *P* < .05 was considered statistically significant. Data were analysed in SAS statistical software (version 9.4; SAS Institute Inc.).

## 3. Results

We identified 40 778 patients with incident UC 2007-2021 and 32 423 patients with prevalent UC as of July 1, 2008. After the exclusion of participants with previous use of immunomodulators or TTs, or contraindication to their use, 63 925 patients remained. The total number of matched population comparator subjects was 593 072 (Table S10).

### 3.1. Characteristics of the study population

Patient characteristics differed among treatment cohorts (Table 1). Patients in the thiopurine + TNFi cohort were the youngest at diagnosis/match (median age 26.7 years) and at start of follow-up (median age 32.2) and had fewer comorbidities. Patients treated with vedolizumab, ustekinumab, and tofacitinib had the largest proportion of extensive disease (67%-72%). Patients in the naïve cohort were oldest (median age at start of follow-up = 46.6 years), more often had comorbidities, and less often extensive disease (31%).

**Table 1.** Characteristics at the start of follow-up in the treatment cohorts: naïve (no treatment with thiopurines, tumor necrosis factors inhibitors (TNFi), and other targeted therapies), thiopurine (treatment with thiopurines), TNFi (treatment with TNFi), thiopurine + TNFi (overlapping treatment with thiopurine and TNFi), and treatment with vedolizumab, ustekinumab, and tofacitinib, in patients without a history of cancer.

	Treatment cohort						
Characteristic	Naïve	Thiopurines	TNFi	Thiopurines + TNFi	Vedolizumab	Ustekinumab	Tofacitinib
Total	49 688	11 916	7209	3452	1618	649	413
*Sex, n (%)*							
Female	24 403 (49.1%)	5159 (43.3%)	3231 (44.8%)	1490 (43.2%)	729 (45.1%)	305 (47.0%)	174 (42.1%)
Male	25 285 (50.9%)	6757 (56.7%)	3978 (55.2%)	1962 (56.8%)	889 (54.9%)	344 (53.0%)	239 (57.9%)
*Age at diagnosis*							
Mean (SD)	41.1 (18.1)	33.0 (16.2)	31.5 (14.4)	29.9 (14.3)	32.7 (16.3)	30.4 (14.4)	31.5 (13.6)
Median (IQR)	38.2 (26.9-54.0)	29.2 (20.6-42.9)	28.3 (20.8-40.0)	26.7 (19.3-38.3)	28.2 (20.2-41.9)	27.6 (20.0-38.4)	28.3 (21.3-39.9)
* Categories, n (%)*							
<18y	3694 (7.4%)	2038 (17.1%)	1157 (16.0%)	705 (20.4%)	267 (16.5%)	120 (18.5%)	61 (14.8%)
18-<40y	22 803 (45.9%)	6404 (53.7%)	4255 (59.0%)	1974 (57.2%)	913 (56.4%)	384 (59.2%)	249 (60.3%)
40-<60y	14 478 (29.1%)	2479 (20.8%)	1447 (20.1%)	626 (18.1%)	293 (18.1%)	113 (17.4%)	87 (21.1%)
≥60y	8713 (17.5%)	995 (8.4%)	350 (4.9%)	147 (4.3%)	145 (9.0%)	32 (4.9%)	16 (3.9%)
*Age at start of follow-up*							
Mean (SD)	47.5 (18.3)	38.2 (17.1)	37.9 (15.6)	35.3 (15.1)	40.8 (17.3)	40.3 (15.6)	40.3 (14.6)
Median (IQR)	46.6 (32.8-61.5)	35.0 (24.7-50.5)	35.2 (25.6-48.8)	32.2 (23.6-45.3)	36.3 (26.8-53.5)	37.3 (27.5-51.0)	36.8 (28.4-49.6)
* Categories, n (%)*							
<18y	1499 (3.0%)	1154 (9.7%)	461 (6.4%)	336 (9.7%)	58 (3.6%)	19 (2.9%)	1 (0.2%)
18-<40y	17 497 (35.2%)	5917 (49.7%)	3859 (53.5%)	1940 (56.2%)	844 (52.2%)	347 (53.5%)	236 (57.1%)
40-<60y	17 011 (34.2%)	3240 (27.2%)	2115 (29.3%)	891 (25.8%)	428 (26.5%)	198 (30.5%)	126 (30.5%)
≥60y	13 681 (27.5%)	1605 (13.5%)	774 (10.7%)	285 (8.3%)	288 (17.8%)	85 (13.1%)	50 (12.1%)
*Education level (years), n (%)*							
≤9	10 277 (20.7%)	2264 (19.0%)	1189 (16.5%)	618 (17.9%)	248 (15.3%)	104 (16.0%)	47 (11.4%)
10-12	22 634 (45.6%)	5476 (46.0%)	3373 (46.8%)	1571 (45.5%)	739 (45.7%)	321 (49.5%)	201 (48.7%)
>12	16 593 (33.4%)	4138 (34.7%)	2629 (36.5%)	1253 (36.3%)	628 (38.8%)	223 (34.4%)	165 (40.0%)
Missing	184 (0.4%)	38 (0.3%)	18 (0.2%)	10 (0.3%)	3 (0.2%)	1 (0.2%)	(0.0%)
*Country of birth*							
Nordic	45 054 (90.7%)	10 547 (88.5%)	6315 (87.6%)	3004 (87.0%)	1415 (87.5%)	560 (86.3%)	375 (90.8%)
Non-Nordic	4632 (9.3%)	1368 (11.5%)	894 (12.4%)	448 (13.0%)	203 (12.5%)	89 (13.7%)	38 (9.2%)
Missing	2 (0.0%)	1 (0.0%)	0 (0.0%)	0 (0.0%)	0 (0.0%)	(0.0%)	(0.0%)
*Co-morbidity past 5 years, n (%)*							
Diabetes mellitus^a^	2761 (5.6%)	542 (4.5%)	299 (4.1%)	132 (3.8%)	94 (5.8%)	40 (6.2%)	17 (4.1%)
Ischemic heart disease^b^	1221 (2.5%)	146 (1.2%)	66 (0.9%)	18 (0.5%)	17 (1.1%)	5 (0.8%)	2 (0.5%)
Hypertension^c^	11 593 (23.3%)	1720 (14.4%)	977 (13.6%)	374 (10.8%)	321 (19.8%)	122 (18.8%)	64 (15.5%)
Chronic obstructive pulmonary disease^d^	249 (0.5%)	39 (0.3%)	15 (0.2%)	6 (0.2%)	6 (0.4%)	2 (0.3%)	1 (0.2%)
Cerebrovascular disease^e^	595 (1.2%)	64 (0.5%)	21 (0.3%)	9 (0.3%)	7 (0.4%)	3 (0.5%)	(0.0%)
Rheumatic diseases^f^	280 (0.6%)	66 (0.6%)	237 (3.3%)	36 (1.0%)	8 (0.5%)	15 (2.3%)	22 (5.3%)
Depression and anxiety^g^	9965 (20.1%)	2107 (17.7%)	1534 (21.3%)	653 (18.9%)	407 (25.2%)	196 (30.2%)	104 (25.2%)
*Medications during past 5 years, n (%)*							
Drugs treating peptic ulcer and reflux	14 859 (29.9%)	5437 (45.6%)	3434 (47.6%)	1660 (48.1%)	825 (51.0%)	340 (52.4%)	202 (48.9%)
Antidiabetics	2877 (5.8%)	595 (5.0%)	336 (4.7%)	142 (4.1%)	100 (6.2%)	44 (6.8%)	20 (4.8%)
Non-steroid anti-inflammatory drugs (NSAIDs)	17 177 (34.6%)	3291 (27.6%)	2084 (28.9%)	877 (25.4%)	371 (22.9%)	156 (24.0%)	104 (25.2%)
Opioids	14 405 (29.0%)	3687 (30.9%)	2722 (37.8%)	1168 (33.8%)	680 (42.0%)	296 (45.6%)	199 (48.2%)
Antihypertensives	12 838 (25.8%)	2030 (17.0%)	1220 (16.9%)	467 (13.5%)	380 (23.5%)	139 (21.4%)	72 (17.4%)
Lipid reducers	6675 (13.4%)	1080 (9.1%)	609 (8.4%)	253 (7.3%)	197 (12.2%)	79 (12.2%)	48 (11.6%)
Antibiotics	31 173 (62.7%)	7740 (65.0%)	4838 (67.1%)	2306 (66.8%)	1118 (69.1%)	466 (71.8%)	270 (65.4%)
Anticoagulants	7918 (15.9%)	1374 (11.5%)	926 (12.8%)	367 (10.6%)	318 (19.7%)	143 (22.0%)	79 (19.1%)
Drugs for obstructive airway diseases	8407 (16.9%)	2073 (17.4%)	1305 (18.1%)	615 (17.8%)	340 (21.0%)	138 (21.3%)	80 (19.4%)
Antidepressants	9501 (19.1%)	2111 (17.7%)	1501 (20.8%)	657 (19.0%)	407 (25.2%)	185 (28.5%)	108 (26.2%)
Anxiolytics	7368 (14.8%)	1652 (13.9%)	1204 (16.7%)	503 (14.6%)	292 (18.0%)	141 (21.7%)	70 (16.9%)
Hypnotics, sedatives	9149 (18.4%)	2316 (19.4%)	1647 (22.8%)	725 (21.0%)	465 (28.7%)	224 (34.5%)	123 (29.8%)
*Montreal Stage at diagnosis/July 1, 2008*							
E1 (ulcerative proctitis)	10 160 (20.4%)	768 (6.4%)	446 (6.2%)	168 (4.9%)	59 (3.6%)	22 (3.4%)	9 (2.2%)
E2 (left side)	10 872 (21.9%)	2747 (23.1%)	1575 (21.8%)	705 (20.4%)	325 (20.1%)	124 (19.1%)	86 (20.8%)
E3 (extensive)	15 343 (30.9%)	6155 (51.7%)	4094 (56.8%)	2031 (58.8%)	1086 (67.1%)	436 (67.2%)	299 (72.4%)
EX (extent not defined)	12 338 (24.8%)	2241 (18.8%)	1088 (15.1%)	548 (15.9%)	148 (9.1%)	66 (10.2%)	19 (4.6%)
Missing	975 (2.0%)	5 (0.0%)	6 (0.1%)	0 (0.0%)	(0.0%)	1 (0.2%)	(0.0%)
*Primary sclerosing cholangitis, n (%)*							
At diagnosis/July 1, 2008	1103 (2.2%)	384 (3.2%)	237 (3.3%)	117 (3.4%)	81 (5.0%)	35 (5.4%)	16 (3.9%)
At the end of follow-up	1900 (3.8%)	598 (5.0%)	339 (4.7%)	177 (5.1%)	104 (6.4%)	39 (6.0%)	18 (4.4%)
*Treatment before first diagnostic listing of ulcerative colitis/July 1, 2008, n (%)*							
5-aminosalicylic acid	37 405 (75.3%)	11 600 (97.3%)	6949 (96.4%)	3353 (97.1%)	1586 (98.0%)	621 (95.7%)	408 (98.8%)
Colectomy	2619 (5.3%)	633 (5.3%)	700 (9.7%)	302 (8.7%)	192 (11.9%)	106 (16.3%)	78 (18.9%)
*Treatment during follow-up, n (%)*							
5-aminosalicylic acid	34 378 (69.2%)	9685 (81.3%)	5103 (70.8%)	2543 (73.7%)	1027 (63.5%)	311 (47.9%)	240 (58.1%)
							
							
							
Colectomy	1374 (2.8%)	980 (8.2%)	626 (8.7%)	382 (11.1%)	170 (10.5%)	51 (7.9%)	24 (5.8%)

^a^≥2 main diagnoses of diabetes mellitus in the National Patient Register or ≥2 redeemed prescriptions for antidiabetic medications in the Prescribed Drug Register.

^b^Hospitalization or ≥2 outpatient visits with a main diagnosis of ischemic heart disease from a Cardiology or Internal Medicine Clinic.

^c^≥2 redeemed prescriptions for antihypertensive medications in the Prescribed Drug Register.

^d^≥2 diagnoses in the National Patient Register.

^e^Hospitalization or ≥2 outpatient visits with a main diagnosis of cerebrovascular disease from a Neurology, Stroke, or Internal Medicine Clinic.

^f^≥2 diagnoses in the National Patient Register.

^g^Hospitalization or ≥2 outpatient visits with a main diagnosis of anxiety or depression in the National Patient Register or ≥2 redeemed prescriptions for antidepressants or anxiolytics in the Prescribed Drug Register.

Abbreviation: TNFI, tumor necrosis factor inhibitor.

Characteristics for patients with incident and prevalent UC are presented separately in Table S11, and detailed characteristics of the treatment cohorts and the number and proportion of patients excluded because of previous cancer are listed in Table S12a and b.

Patients starting in the naïve cohort largely remained within that cohort (86%). Approximately one-fourth (23%) of the patients starting in the thiopurine cohort were later followed in the thiopurine + TNFi cohort, and 36% of patients starting in the TNFi cohort were later followed in the thiopurine + TNFi cohort (Figure S2). The median follow-up was 11.7 years in the naïve cohort, 6.99 years in the thiopurine, 4.3 years in the TNFi, and 5.1 years in the thiopurine + TNFi, 2.8 in the Vedolizumab, 1.6 years in the Ustekinumab, and 1.7 years in the Tofaniticib cohort (Table S13a and b).

### 3.2. Cumulative cancer incidence

**Figure 1.**
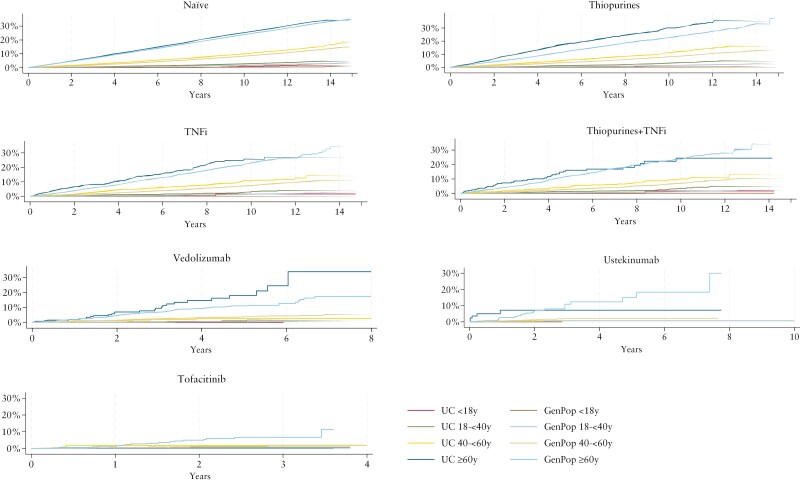
Time to any cancer in cohorts of patients with UC, stratified by age (<18y, 18-<40y, 40-<60y, ≥60y) and treatment at start of follow-up: naïve (no treatment with thiopurine, tumor necrosis factor inhibitors [TNFi], and other targeted therapies), thiopurine (treatment with thiopurines), TNFi (treatment with TNFi), thiopurine + TNFi (overlapping treatment with thiopurines and TNFi), and treatment with vedolizumab (up to 8 years of follow-up), ustekinumab (up to 8 years), and tofacitinib (up to 4 years of follow-up).

During a total follow-up of 5 800 089 person-years, 52 759 cancer events in 656 997 (63 925 + 593 072) participants were registered in the 7 partly overlapping treatment cohorts. The cumulative 5-year incidence of any cancer (for all age groups combined) was 5.3% in the naïve cohort vs 4.1% in its matched general population comparator group, 4.3% in the thiopurine cohort (vs 2.5%), 3.6% in TNFi cohort (vs 2.1%), 3.4% in thiopurine + TNFi cohorts (vs 1.9%), 3.9% in the Vedolizumab cohort (vs 2.6%), 1.4% in the Ustekinumab cohort (vs 2.4%), and 1.0% in the Tofaniticib cohort (vs 2.7%) (Table S13a and b). When stratifying by age groups, the cumulative incidence of any cancer was highest for patients with UC ≥ 60 years and their population comparators, and lowest for participants < 18 years (Figure 1).

### 3.3. Incidence rate differences and HRs for cancer overall

**Figure 2.**
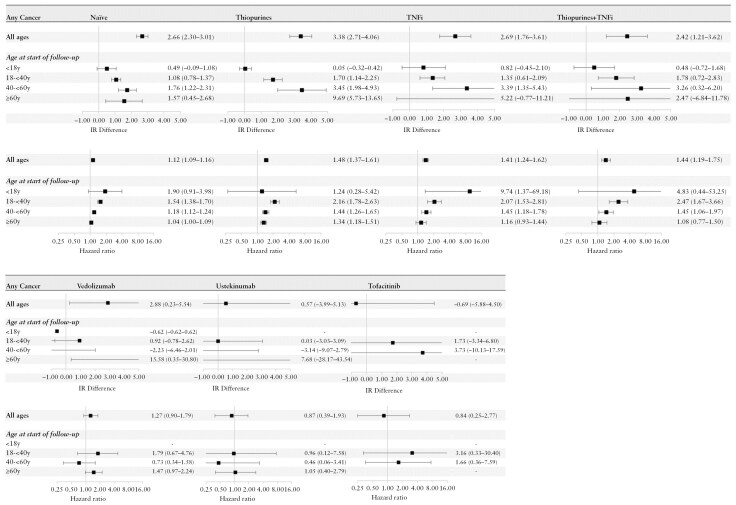
Incidence rate (IR) differences (cases/1000 person-years) and hazard ratios with 95% CIs of any cancer in cohorts of patients with ulcerative colitis vs matched general population comparators, stratified by age (<18, 18-<40, 40-<60, ≥60 years) and treatment at start of follow-up: naive (no treatment with thiopurine, tumor necrosis factor inhibitors (TNFi), and other targeted therapies), thiopurines (treatment with thiopurines), TNFi (treatment with TNFi), thiopurine + TNFi (overlapping treatment with thiopurines and TNFi), and treatment with vedolizumab, ustekinumab, and tofacitinib.

The IR difference vs the population for any cancer was 2.66 cases/1000 person-years in the naïve cohort, 3.38 in the thiopurine, 2.69 in the TNFi, 2.42 in the thiopurine + TNFi cohort, and 2.88 in the vedolizumab cohort, with overlapping CIs. The cohorts treated with ustekinumab and tofaniticib had few cancer events and large CIs that were not significantly increased (Figure 2).

The IR differences were not significantly elevated in pediatric patients (age < 18 years), while the IR differences for adult patients were more pronounced, especially for middle-aged (40 to <60) and elderly (≥60 years) patients, for example, 1.76 to 1.57 cases/1000 years in the naïve cohort, and 3.45 to 9.69 cases/1000 years in the thiopurine cohort. The contrary was observed for the HRs which were increased in younger patients (18 to <40 years) are rarely in patients ≥ 60 years.

### 3.4. Incidence rate differences and HRs by cancer type

**Figure 3.**
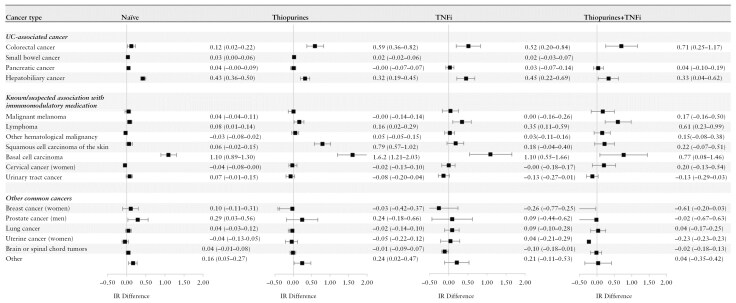
Incidence rate (IR) differences (cases/1000 person-years) and hazard ratios with 95% CIs of UC-associated cancers, cancers with known/suspected association with immunomodulatory, treatment, and cancers common in the population in cohorts of patients with ulcerative colitis vs matched general population comparators, stratified by treatment at the start of follow-up: naive (no treatment with thiopurine, tumor necrosis factor inhibitors (TNFi), and other targeted therapies), thiopurines (treatment with thiopurine), TNFi (treatment with TNFi), and thiopurine + TNFi (overlapping treatment with thiopurine and TNFi). UC, ulcerative colitis.

The IR differences were elevated in the naive cohort and in cohorts treated with TNFi and/or thiopurine for CRC, hepatobiliary cancer, and lymphoma, although lower estimates were observed in the naïve group for CRC and lymphoma (Figure 3, Table S14a). The IR difference for CRC was between 0.12 cases/1000 years in the naïve group and 0.52 to 0.71 cases in patients treated with thiopurines and/or TNFi. The IR difference was 0.32 to 0.45 cases/1000 years for hepatobiliary cancer, 0.08 to 0.61 cases/1000 years for lymphoma, and 0.77 to 1.62 cases/1000 years for basal cell carcinoma. In the cohorts treated with vedolizumab, ustekinumab, and tofacitinib events were few for each cancer type (Figure S3, Table S14b). We observed no significantly increased IRs for common cancer types such as breast, lung, and uterine cancer in any of the treatment cohorts.

**Figure 4.**
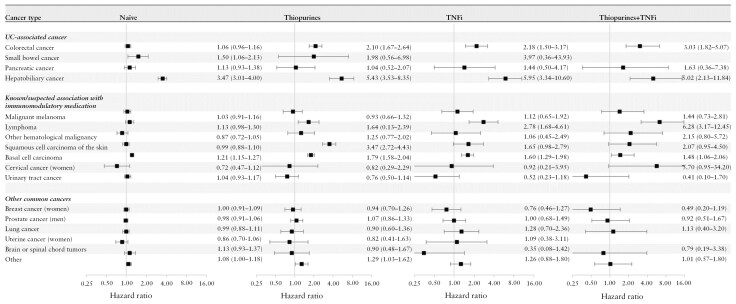
Hazard ratios with 95% CIs of UC-associated cancers, cancers with known/suspected association with immunomodulatory treatment, and cancers common in the population in cohorts of patients with ulcerative colitis vs matched general population comparators, stratified by age (<18, 18-<40, 40-<60, ≥60 years), and treatment at the start of follow-up: naive (no treatment with thiopurine, tumor necrosis factor inhibitors (TNFi), and other targeted therapies), thiopurine (treatment with thiopurines), TNFi (treatment with TNFi), and thiopurines + TNFi (overlapping treatment with thiopurine and TNFi). UC, ulcerative colitis.

The highest HRs compared to the general population were found for hepatobiliary cancer: naïve 3.47; thiopurine 5.43; TNFi 5.95; thiopurine + TNFi 5.02 (Figure 4). When stratifying patients by presence of PSC, the IR differences were more similar between treatment cohorts: PSC naïve cohort 6.26 extra cases; Non-PSC naive cohort 0.28 extra cases (Table S15).

For CRC, the HR was not significantly elevated in the naïve cohort, but in the cohorts treated with thiopurine and/or TNFi (ranging from 2.10 to 3.03). Basal cell carcinoma HRs were elevated in the treatment naive (1.21) and in cohorts treated with thiopurine and/or TNFi (1.48 to 1.79). The HR for lymphoma was elevated in the thiopurine cohort (1.64), TNFi (2.78), and thiopurine + TNFi (6.28), but not in the naïve cohort. No elevated HRs were observed for malignant melanoma, urinary tract cancer, cervical cancer, breast, uterine, prostate, lung, or brain or spinal cord cancers in UC patients vs the general population cohort, regardless of treatment exposure. Hazard ratios for patients treated with vedolizumab, ustekinumab, and tofacitinib are in Figure S4.

## 4. Discussion

In this cohort study including more than 60 000 patients with UC followed for a median of 8.1 years, we observed elevated cancer incidences compared to the general population in patients treated with thiopurine (3.38 additional cases/1000 patients and year), TNFi (2.69 additional cases), thiopurines + TNFi (2.42 additional cases), and vedolizumab (2.88 additional cases). The IRs differences were not increased in cohorts treated with ustekinumab and tofacitinib but included fewer patients. Importantly, patients naïve to immunomodulatory drugs also had an elevated cancer incidence in the order of 2.66 additional cases/1000 years. As expected, we observed large differences across age strata, with the highest increase in absolute risk among middle-aged and elderly patients. Finally, we observed elevated incidences for CRC, hepatobiliary cancer, lymphoma, and basal cell carcinoma, but no risk increases for other common cancer types in any of the treatment cohorts.

A recent Danish study reported an increased risk of cancer in patients with IBD following thiopurine use with or without TNFi.^40^ The study reported an adjusted HR of 1.59 for cancer in patients with IBD < 50 years of age vs reference population, which aligns well with our results of HR 1.54 in naïve patients 18 to <40 years. The Danish study did not report absolute risk estimates in relation to the population, but focused on HRs, which were adjusted for several factors, including socio-economic status and disease severity, thus reflecting the relative risk increase from IBD and IBD medication.

### 4.1. Cancers associated with UC

We observed elevated incidences of certain gastrointestinal cancer types associated with UC, for example, CRC and hepatobiliary cancer. In clinical practice, treatment choices often follow a step-up approach guided by the severity of inflammation, and chronic inflammation is an important driver of cancer risk. Patients requiring thiopurine and/or TNFi treatment (likely due to extensive and more active disease) had a higher incidence of CRC (0.71 additional case of CRC per 1000 person-years in patients needing combined treatment with thiopurine and TNFi) than IBD-free individuals of the same age and sex in the general population cohort. A meta-analysis investigating risk factors and protective factors for the CRC development in patients with IBD has reported a pooled odds ratio of 0.55 (95% CI, 0.37-0.82) for thiopurine use (according to 19 studies), and an odds ratio (OR) of 0.71 (95% CI, 0.14-3.67) for TNFi use (according to 4 studies),^19^ suggesting protective effects of these medications.

We observed elevated IR differences and HRs for hepatobiliary cancer vs the matched population comparators, although its cumulative incidence was low (0.5% after a median 11.7 years of follow-up), compared to several other cancer types. A meta-analysis based on 7 studies reported an IR ratio of 2.05 (95% CI, 1.52-2.76) for hepatobiliary cancer in patients with UC compared to the general population.^11^ Primary sclerosing cholangitis has been identified as a risk factor for death from hepatocellular carcinoma and cholangiocarcinoma,^10^ but with no excess risk reported from thiopurine of TNFi exposure.^41^ In our study, the prevalence of PSC was lower in the naïve cohort (3.8% at end of follow-up) than in cohorts treated with thiopurine and/or TTs (4.4%-6.4%), and the HRs for hepatobiliary cancer were similar between the treatment cohorts when stratifying for presence of PCS.

Inflammatory bowel disease has been reported to be associated with small bowel cancer,^42^ but these are rare, and in the present study no cancer events occurred in the TNFi and thiopurine + TNFi cohorts. Although pancreatic cancer has been found to be slightly more prevalent in patients with than without IBD,^43^ it has not been associated with immunomodulators or TTs.

### 4.2. Extraintestinal cancers

A previous meta-analysis reported an IR ratio of 1.15 (95% CI, 1.02-1.31) for extraintestinal cancers overall in UC patients (irrespective of UC medication status) compared to the general population.^11^ We observed increased incidences of basal cell carcinoma, squamous cell carcinoma, and lymphoma. Lymphoma incidence was increased only in the groups treated with thiopurine and/or TNFi, which is in line with previous reports.^20,44,45^ The findings of increased incidence of basal cell carcinoma across all treatment groups also align with previous reports.^46^

### 4.3. Strengths and limitations

This study was enabled by the availability of prospectively recorded data from registers providing virtually complete follow-up for routine medical practice. Because of the population-based setting, our results should be highly generalizable to similar populations. Previous studies have assessed cancer diagnoses according to organ site by using diagnostic codes. To enhance the specificity of a cancer diagnosis, we also required histopathologic confirmation from the Cancer Register. The 1-year lag time decreased detection bias, because cancer events during the first year following UC diagnosis were not considered events.

The study has several limitations. In studying cancer as an outcome, long follow-up is preferable. However, to ensure that all included patients were new users, that is, naïve to thiopurine/TNFi therapy, we did not include prevalent patients earlier than 3 years after the start of the Prescribed Drug Register. Our median follow-up was 8 years, and we know from previous research that CRC risk increases after 8-10 years after diagnosis. Our results must therefore be interpreted considering limited follow-up time. In addition, follow-up time was even shorter for the patients receiving the newer TTs tofacitinb (7.3 years) and vedolizumab (6.2 years). The newer TTs were approved 2014-2019, that is, during a time when treatment has become more proactive, individualized, and when cancer surveillance has intensified.

We were able to present estimates of cancer risk among treatment-naïve patients as a comparison group, but this study was not designed to compare the treatment groups. The age and sex distributions in each of the treatment cohorts vs the general population cohorts were controlled through the matching, but pronounced differences existed in, for example, age and disease severity between the different treatment groups, thus precluding comparison of incidence across treatment groups. We used a once-exposed, always-exposed approach and did not consider actual exposure time.

### 4.4. Clinical implications

The aim of this study was to provide an overview of cancer risk among patients treated with modern IBD medications. Our design answered the following question: What is the absolute risk of cancer in patients with IBD requiring immunomodulatory treatment, and the relative risk with respect to individuals without IBD and associated treatment? The question whether advanced treatments differ with regard to cancer risk after careful adjustment for relevant confounders is another question, that we did not address herein.

Colorectal cancer surveillance programs are an established component of health care for patients with IBD.^47^ In the general population in Sweden, the program Screening of Swedish colons, targeting individuals aged 59-62 years, was initiated in 2014. Screening of patients with IBD for other forms of cancer, such as hepatobiliary cancer and basal cell skin carcinoma, has been suggested. For non-melanoma skin cancer, Sweden has no national screening recommendations, and the overall absolute risks were low. Therefore, evidence remains insufficient to support specific surveillance strategies. Reassuringly, elevated risks were not observed for common cancers such as breast, prostate, and lung cancer, regardless of treatment status.

## 5. Conclusion

Comparing UC to the general population, the additional risk of developing cancer amounts to 2 to 3 extra cases of cancer per 1000 years, also in patients naïve to immunomodulatory drugs. This additional risk displayed large variations with age but—once age and sex were considered—relatively small differences between different UC therapies.

## Supplementary Material

jjaf091_suppl_Supplementary_Tables_S1-S15_Figures_S1-S4

## Data Availability

The data analyzed for this article cannot be shared publicly, because of Swedish legislation.
