# Supplementary material for: Cancer incidence in patients with ulcerative colitis naïve to or treated with thiopurine and targeted therapies—a cohort study 2007 to 2022 with comparison to the general population
Source: J Crohns Colitis. 2025 Jun 2;19(6):jjaf091. doi: 10.1093/ecco-jcc/jjaf091 (PMC12203221; doi:10.1093/ecco-jcc/jjaf091)
Supplement: jjaf091_suppl_Supplementary_Tables_S1-S15_Figures_S1-S4 [file jjaf091_suppl_supplementary_tables_s1-s15_figures_s1-s4.pdf]

Supplement to: Cancer Incidence in Patients with Ulcerative Colitis Naïve to or Treated with Thiopurine and Targeted Therapies– a cohort study 2007 to 2022 with comparison to the general population

Åsa H Everhov, Johan Askling, Jonas Söderling, Jonas Halfvarson, Julia Eriksson, SWIBREG study group*, Karin E Smedby, Jonas F Ludvigsson, Henrik Toft Sørensen, Ola Olén

*Collaborators: Hans Strid, Henrik Hjortswang, Malin Olsson, Johann Hreinsson, Charlotte Hedin, Jonas L Bengtsson, Marie A Andersson, Pontus Karling, Martin Rejler, Susanna Jäghult, Ulrika Fagerberg, Pär Myrelid, Caroline Nordenvall, Karl Mårild

Table of Contents

Table S1. Meta-analyses published from year 2014, and cohort studies from 2020 and onwards describing cancer risk with thiopurine and tumor necrosis factor inhibitors (TNFi) treatment, and population-based cohort studies published from year 2020 reporting cancer incidence in thiopurine/TNFi-treated patients 4

Table S2. Overview of data sources 6

Table S3. Codes defining ulcerative colitis (UC) and inflammatory bowel disease (IBD)...... 7

Table S4. Exclusion criteria, defined as any registration before start of follow-up...... 8

Table S5. Drug exposure definitions in Swedish National Patient Register, the Swedish Prescribed Drug Register, and the Swedish quality register for inflammatory bowel disease (SWIBREG)...... 9

Table S6. Codes in the Swedish Cancer Register used to classify cancer types 10

Table S7. ICD (only main diagnoses) - and ATC codes used to define comorbidity...... 12

Table S8. Codes for medication in the Prescribed Drug Register...... 13

Table S9. Nordic Medical Statistics Committee (NOMESCO) Classification of Surgical Procedures codes associated with inflammatory bowel disease...... 14

Table S10. Flowchart of identified patients and comparators 16

Table S11. Characteristics of incident patients with UC from January 1st, 2007 to December 31st, 2021, and prevalent patients with UC as of July 1st 2008 and matched population comparators...... 17

Table S12a. Baseline characteristics of patients with UC and matched population comparators at start of follow-up in naïve patients and patients treated with THFi and/or thiopurine 21

Table S12b. Baseline characteristics of patients with UC and matched population comparators at start of treatment with vedolizumab, Ustekinumab, and Tofacitinib 24

Table S13a. Number, n, and proportion (%) of events of ulcerative colitis (UC)-associated cancers, cancers with known/suspected association with immunomodulatory treatment, and cancers common in the population during follow-up in patients with UC, stratified by treatment at start of follow-up: naive (no treatment with thiopurine, tumor necrosis factor inhibitors (TNFi) and other targeted therapies), thiopurines (treatment with thiopurines), TNFi (treatment with TNFi), and thiopurines+TNFi (overlapping treatment with thiopurine and TNFi)........ 27

Table S13b. Number, n, and proportion (%) of events of ulcerative colitis (UC)-associated cancers, cancers with known/suspected association with immunomodulatory treatment, and cancers common in the population during follow-up in patients with UC, stratified by treatment at start of follow-up: vedolizumab, ustekinumab, and tofacitinib 29

Table S14a. Incidence rate (cases/1000 person years) and IR differences of UC-associated cancers, cancers with known/suspected association with immunomodulatory treatment, and cancers common in the population different cancers up in cohorts of patients with UC, stratified by treatment at start of follow-up: naive (no past or ongoing treatment with thiopurines, tumor necrosis factor inhibitors (TNFi) and other targeted therapies), thiopurine (treatment with thiopurine), TNFi (treatment with TNFi), and thiopurine+TNFi (overlapping treatment with thiopurine and TNFi) 31

Table S14b. Incidence rate (cases/1000 person years) and IR differences of UC-associated cancers, cancers with known/suspected association with immunomodulatory treatment, and cancers common in the population different cancers up in cohorts of patients with UC, stratified by treatment at start of follow-up: vedolizumab, Ustekinumab, and tofacitinib 33

Table S15. Hazard ratios and incidence rate differences, stratified by presence of primary biliary cirrhosis (PSC) at baseline 34

Figure S1. Overview of timeline for approval of biologic drugs in Sweden........ 36

Figure S2. Number of patients remaining within the same treatment cohort or starting follow-up in another treatment cohort:........ 37

Figure S3. Incidence rate (IR) differences (cases/1000 person years) and hazard ratios with 95% confidence intervals (Cis) of of UC-associated cancers, cancers with known/suspected association with thiopurine treatment, and cancers common in the population in cohorts of patients with ulcerative colitis versus matched general population comparators, stratified by treatment at start of follow-up: vedolizumab, ustekinumab, and tofacitinib........ 39

Figure S4. Hazard ratios with 95% confidence intervals (Cis) of of UC-associated cancers, cancers with known/suspected association with immunomodulatory treatment, and cancers common in the population in cohorts of patients with ulcerative colitis versus matched general population comparators, stratified by treatment at start of follow-up: vedolizumab, ustekinumab, and tofacitinib........ 40

References 41

Table S1. Meta-analyses published from year 2014, and cohort studies from 2020 and onwards describing cancer risk with thiopurine and tumor necrosis factor inhibitors (TNFi) treatment, and population-based cohort studies published from year 2020 reporting cancer incidence in thiopurine/TNFi-treated patients

	Included studies/patients	Exposure	Comparison group	Outcome	Main result
**Meta-analyses based on randomized controlled trials (2014-)**					
Williams, APT 2014 [1]	22 RCT (15 in CD, and 6 in UC)	TNFi	Placebo	Any cancer	The relative risk of malignancy was not significantly different between the treatment and placebo groups (0.77; 95% CI 0.37–1.59) during a mean follow-up of 205 days
Bonovas, CGH, 2016 [2]	23 RCTs	Biologics	Placebo	Any cancer	Use of biologics was not associated with increased cancer risk (OR, 0.90; 95% CI, 0.54–1.50) during a follow-up of 1 to 24 months
Bezzio, Cancers, 2023 [3]	26 studies (22 RCTs)	Tofacitinib	Placebo	Overall cancer, non-melanoma skin cancer, lung cancer, breast cancer, and cervical cancer	The risk of developing cancer during therapy with tofacitinib was similar to that in patients treated with a placebo or other drugs (RR = 1.06; *p* = 0.57) during a follow-up of 2 to 38.5 months
Russel [4]	62 phase II, III or IV RCTs and 16 long-term extension studies	JAK-inhibitors	Placebo	Any cancer	Incidence rate ratio between JAKi and placebo: 0.71; 95%CI 0.44 to 1.15 between JAKi and TNFi: 1.50; 95%CI 1.16 to 1.94 during a mean follow-up of 118 weeks
**Meta-analyses based on observational studies (2014-)**					
Piovani, APT 2020 [5]	9 studies of patients >60 years with IBD	Biologics	Patients with IBD not treated with biologics	Any cancer	Frequency 0.67% in the groups exposed to biologics and 0.74% in the unexposed, RR: 0.90; 95% CI: 0.64-1.26
Borren, CGH 2019 [6]	14 studies on patients < 60 years with immune-mediated diseases (IBD, RA, psoriasis)	Biologics	Patients with immune-mediated disease not treated with biologics	Any cancer	OR 0.54, 95% CI, 0.28–1.05 vs no use
Elmahdi, JNO 2022 [7]	2 studies of pediatric-onset IBD	Thiopurines	Population	Any cancer	RR 2.09; 95% CI 1.55-2.83 in thiopurine exposed and 1.82; 95% CI 0.63-5.22 in patients not exposed vs population
Zhu JCC 2018 [8]	27 observational studies (11 cohort, 16 case-control, 10 UC)	Thiopurines	Patients with IBD not treated with thiopurines	Colorectal cancer	Case-control studies: **OR = 0.49, 95% CI: 0.34–0.70** for thiopurine use<br>Cohort studies: **RR = 0.96, 95% CI: 0.94–0.98**
Lu APT 2018 [9]	24 observational studies (12 UC)	Thiopurine	Patients with UC not treated with thiopurines	Colorectal cancer	OR in UC = **0.67, 95% CI 0.45-0.98** for thiopurines vs no thiopurine treatment
Jess, 2014 [10]	15 studies (13 clinic-based, 2 population-based)	Thiopurine	Patients with IBD not treated with thiopurines	Colorectal cancer or high-grade dysplasia	OR 0.87; 95% CI, 0.71–1.06 vs no exposure
Wijnards, Gastro 2021 [11]	164 studies	Multiple risk factors		Colorectal cancer or high grade dysplasia	Moderate evidence for thiopurines as a protective factor in univariable analysis
Chupin, APT 2020 [12]	4 observational studies	TNFi and/or thiopurines	Patients with IBD not treated with thiopurines and/or TNFi	Lymphoma	Incidence rate ratio for thiopurines monotherapy: **2.23 (95% CI: 1.79-2.79);** combination therapy: **2.49 (95% CI: 1.39-**

					**4.47**, TNFi monotherapy **1.52 (95% CI: 1.06-2.19);** compared to no exposure to anti-TNF or thiopurines
Yang, JCC 2018 [13]	12 studies on patients with IBD	TNFi	Patients with IBD not treated with TNFi	Lymphoma	Pooled crude incidence rates 5.40 cases per 10000 patient-years [95% CI, 4.22–6.59] in patients exposed to TNFi and 4.25 cases per 10000 patient-years [95% CI, 3.95–4.54] in patients unexposed
Kotlyar, CGH 2015 [14]	8 population-based and 10 referral studies of patients with IBD	Thiopurine	Current, former, never use	Lymphoma	**SIR = 5.71; 95% CI, 3.72–10.1 in current users**<br>SIR = 1.42; 95% CI, 0.86–2.34 former users<br>SIR=1.06 (95% CI, 0.81–1.40) for never use
Singh, CGH, 2014 [15]	2 studies on patients with IBD	Thiopurine or NNFi	Patients with IBD not treated with thiopurines or TNFi	Melanoma	TNFi: RR 1.08; 95%CI 0.59-1.96<br>Thiopurine: RR 1.10; 95%CI 0.73-1.66) vs non-use
Esse, JAMA Derm, 2020 [16]	2 studies of patients with IBD	Biologics	Patients treated with conventional systemic therapy	Melanoma	RR 1.20 (95% CI, 0.60-2.40) vs biologics-naive
Huang, JGH 2019 [17]	13 studies (3 nested case-control, 10 cohort studies)	Thiopurines	Patients with IBD not treated with thiopurines	Skin cancer	Skin cancer: **RR 1.80 (95% CI 1.14–2.87)** vs no use<br>NMSC: **1.88 (95% CI 1.48–2.38)**<br>Melanoma: 1.22 (95% CI 0.90–1.65)
Ariyaratnam AJG 2014 [18]	8 studies on patients with IBD	Thiopurine	Patients not treated with thiopurines	Non-melanoma skin cancer	Pooled adjusted HR **2.28 (95% CI: 1.50 to 3.45)** vs no use
Kim, SJG 2023 [19]	3 studies included immunomodulator users and 15 undefined users	Immunomodulators	Population	Cervical cancer	Pooled RR in immunomodulators users 2.18 (95% CI, 0.81–5.87) and 1.34 (95% CI, 1.07–1.69) in the non-defined group
Mann, CTG 2022 [20]	5 studies of patients with IBD	TNFi or thiopurines	Population	Cervical cancer	TNFi HR: 1.19; 95% CI: 0.64–2.21 Thiopurines: HR: 0.96; 95% CI: 0.60–1.50
**Cohort studies (2020-) reporting IR or SIR**					
Yu, BMJ Open [21]	131,492 patients with IBD	Thiopurines +/- TNFi	General population	Lymphoma	Thiopurines alone: SIR 2.8; 95% CI 1.4 to 5.7; Thipurines+ anti-TNF-α agents: SIR 5.7; 95% CI 2.7 to 11.9
Charkaoui, JCC 2022 [22]	32,403 patients with UC	TNFi	Patients with UC not treated with TNFi	Colorectal cancer	Unexposed: IR: 1.32 (1.14-1.52)<br>Exposed IR: 1.04 (0.80-1.35)
Ardabili, APT 2022 [23]	1,016 patients with IBD	Thiopurine	No	Any cancer	Non-melanoma skin cancer IR 3.33 (1.97–5.30)<br>Lymphoma IR 1.04 (0.38–2.31)<br>Urinary tract cancer IR 0.21 (0.01–1.03)

RR, relative risk; OR, odds ratio; CI, confidence interval; IBD, inflammatory bowel disease; UC, ulcerative colitis, RA, rheumatoid arthritis; TNFi, tumor necrosis factor inhibitor

Table S2. Overview of data sources

Register name	Administrator	Type of data	Start year
Total Population Register [24]	Statistics Sweden	Data on all persons registered in Sweden, including birth date, sex, place of residence (parish, municipality, or county, depending on availability of reference individuals), last immigration and emigration dates, and identity of parents/siblings	1968-
Longitudinal Integrated database for health insurance and labour market studies (LISA) [25]	Statistics Sweden	Type of work, compensations from employment, entrepreneurial activities, studies, national military service, illness, parental leave, unemployment, labour market activity, rehabilitation, partial retirement, early retirement, retirement, occupational pension, annuities, social assistance, private pensions, country of birth and parental countries of birth, place of employment, highest level of education	1990-
Swedish National Patient Register [26]	Swedish National Board of Health and Welfare	Data from outpatient and inpatient visits in hospital-based care, including date of visit, primary and secondary diagnoses and procedures	1964- hospitalizations<br>1997- outpatient surgery<br>2001- specialized outpatient visits
Swedish quality register for inflammatory bowel diseases SWIBREG [27]		Date of IBD diagnosis, Montreal stage, complications, age at diagnosis, and start- and stop-date of biologic treatment	2005-
Prescribed Drug Register [28]	Swedish National Board of Health and Welfare	Names and Anatomical Therapeutic Chemical (ATC) Classification of prescribed drugs dispensed from pharmacies. The coverage of the register is complete for prescriptions in ambulatory care (both specialist and primary care), while in-hospital drug treatment is generally not recorded on a patient level.	2005-

Table S3. Codes defining ulcerative colitis (UC) and inflammatory bowel disease (IBD)

		ICD-7	ICD-8*	ICD-9	ICD-10
		**1964-1968**	**1969-1986**	**1987-1996**	**1997-**
Inflammatory bowel disease (IBD)	Ulcerative colitis (UC)	572,20; 572,21; 578,03	569,04 563,1; 563,10; 569,02	556	K51
	Crohn's disease (CD)	572,00; 572,09	563,00	555	K50
	Inflammatory bowel disease unclassified (IBD-U)	UC+CD	UC+CD Or 563; 563,0; 563,9; 563,98; 563,99	UC+CD	UC + CD, or K52.3

Table S4. Exclusion criteria, defined as any registration before start of follow-up

	ICD-7	ICD-10	Procedure code	ATC
Any cancer in situ or invasive cancer[1]	140-205	C00-C97		
Human immunodeficiency virus (HIV)[2]		B20-B22		
Chronic hepatitis[2]		B18, K73		
Chronic liver disease[2]		K72, R18, I85		
Transplantation[2]		Z94	DR008, DR010	
Chronic kidney disease (glomerulonephritis, dialyisis, kidney transplantation)[2]		N18.3-N18.5, N03, N05, Z49.1, Z49.2, Z99.2	KAS00, KAS10, KAS20	
Immunomodulator[3]				L04AX03/L01BA01, L01BB02, L04AX01
Tumor necrosis factor inhibitor (TNFi)[3]				L04AB02 (L04AA12 before 2008)<br>L04AB04 (L04AA17 before 2008)<br>L04AB06
Vedolizumab[3]				L04AA33
Ustekinumab[3]				L04AC05
Tofacitinib[3]				L04AA29

[1] in the Cancer Register, [2] in the National Patient Register, [3] in the Prescribed Drug Register

Table S5. Drug exposure definitions in Swedish National Patient Register, the Swedish Prescribed Drug Register, and the Swedish quality register for inflammatory bowel disease (SWIBREG)

Drug group	Substance	ATC-code
**Targeted therapies**		
**Anti-TNFα**	Infliximab	L04AB02 (L04AA12 before)
	Adalimumab	L04AB04 (L04AA17 before)
	Golimumab	L04AB06
**α4β7 integrin-inhibitor**	Vedolizumab	L04AG05 (L04AA33 before 2008)
**Inhibitors of interleukin 12 and 23**	Ustekinumab	L04AC05
**Janus kinase inhibitor**	Tofaniticib	L04AA29
**Thiopurine**		
	Azathioprine	L04AX01
**Aminosalicylates (5-ASA)**		
	Sulfasalazine	A07EC01
	Mesalazine	A07EC02
	Olsalazine	A07EC03
	Balsalazide	A07EC04

Table S6. Codes in the Swedish Cancer Register used to classify cancer types

For morphological codes in accordance with C24, the behavior/malignancy corresponds to the third digit of the code, *i.e*, 5. malignant tumor, infiltrating but rarely metastasizing (*e.g*., basal cell carcinoma), and 6. malignant tumor, infiltrating and usually metastasizing. We defined *any cancer* as a cancer with an ICD-10 code of C00-C97 in combination with a histopathological code in the Cancer Register ending in 5 or 6, (with certain exceptions, as described in the Cancer Coding Instructions.[29]

Location	ICD-7 (1958-)	OR	ICD-10 (1993-)	AND	PAD (1958-)
All cancer	140-207		C00-C97		XX5 or XX6, 441
	193		C70- C72, C751, C753		any
	195, 192.1		-		any
	175, 176.9				051, 063, 053
	180.1, 181				any
	192				461
			D46		223
			D47.3		293
(Incl basal cell carcinoma)					Separate register
**IBD-associated cancer**					
Colorectal cancer	153-154		C18-C20		XX5 or XX6
Small bowel cancer	152		C17		XX5 or XX6
Pancreatic cancer	157		C25		XX5 or XX6
Hepatobiliary cancer	155,156		C22-C24		XX5 or XX6
**Known/suspected association with thiopurines**					
Malignant melanoma	190		C43		176
Lymphoma (incl chronic lymphatic leukemia)	200-202, 2041		C81-86, C88, C911, C913, C914, C91.6, C918		XX5 or XX6
Other hematological malignancy	2024, 203, 2040, 2044, 2049, 205-207		C90-C95 (except C911, C913, C914, C916, C918)		XX5 or XX6
			D46		223
Squamous cell carcinoma of the skin	191		C44		146
Basal cell carcinoma					Separate register
Cervical cancer (women)	171		C53		XX5 or XX6
Urinary tract cancer	180-181		C64-C68		XX5 or XX6
	180.1, 181				any
**Common cancers**					
Breast cancer (women)	170		C50		XX5 or XX6
Prostate cancer (men)	177		C61		XX5 or XX6
Lung cancer	162		C34		XX5 or XX6
Uterus (women)	172, 174		C54-C55		XX5 or XX6
Brain and spinal chord tumors	193		C70- C72, C751, C753		any

Other	140-151, 158-161, 163-169, 171, 173, 175-176,178-179, 182-189, 192, 194-199		C00-C16, C21, C26-C33, C35-C42, C45-C49, C51-C53, C56-C60, C62-C63, C69, C73-C80, C87, C89, C96-C97 **Not** C751, C753, C911		XX5 or XX6
	175, 176.9				051, 063, 053
	192				461
	192.1				any
	195, 192.1		-		any
	175, 176.9				051, 063, 053
			D47.3		293

Table S7. ICD (only main diagnoses) - and ATC codes used to define comorbidity

Comorbidity	ICD-code	ATC-code
Ischemic heart disease	I20-I25	
Chronic obstructive pulmonary disease	J41-J44	
Cerebrovascular disease	I60-I69	
Rheumatic diseases:		
Rheumatoid arthritis	M05, M060, M062, M063, M068, M069, M123	
Ankylosing spondylitis	M45	
Psoriasis arthritis	L405, M070, M071, M073	
Systemic lupus erythomatosus	M320, M321, M328, M329	
Diabetes mellitus	E10-14, O24	A10
Hypertension		C02, C03, C07, C08
Depression and anxiety	F32, F33, F41	N06A, N05B

For ischemic heart disease we required either a hospitalization or minimum 2 outpatient visits at a cardiology or internal medicine clinic, and for cerebrovascular disease either a hospitalization or minimum 2 visits at a neurology or internal medicine clinic. A diagnosis of rheumatic disease and chronic obstructive pulmonary disease required minimum 2 diagnoses (in- or outpatient visits). For diseases diabetes mellitus and depression and anxiety, we used either 2 diagnoses in the National Patient Register or 2 dispensings in the Prescribed Drug Register for a related medication. A diagnosis of hypertension required minimum 2 dispensings of antihypertensive medication in the Prescribed Drug Register

Table S8. Codes for medication in the Prescribed Drug Register

	ATC-code
Drugs against peptic ulcer and reflux	A02A, A02B
Antidiabetics	A10
Non-steroid anti-inflammatory drugs (NSAID)	M01A
Opioids	N02A
Antihypertensives	C02, C03, C07, C08, C09
Lipid reducers	C10
Antibiotics	J01
Anticoagulants	B01
Drugs for obstructive airway diseases	R03
Antidepressants	N06A
Anxiolytics	N05B
Hypnotics, sedatives	N05C

Table S9. Nordic Medical Statistics Committee (NOMESCO) Classification of Surgical Procedures codes associated with inflammatory bowel disease

Surgical procedure	7th revision (KKÅ97)
**Colectomy**	
Subtotal colectomy with end ileostomy	
Colectomy and ileostomy, with closure of the rectum	JFH10
Laparoscopic colectomy and ileostomy	JFH11
Other colectomy	JFH96
Colectomy with IRA (ileorectal anastomosis)	
Colectomy with with ileorectal anastomosis	JFH00
Laparoscopic colektomy with ileorektal anastomosis	JFH01
Ileorektal anastomosis	JFC40
Laparoscopic ileorectal anastomosis	JFC41
Closure of enterostomy with anastomosis to the rectum	JFG29
Closure of enterostomy with anastomosis to colon	JFG26
Partial colectomies	
Right-sided colectomy	JFB30, JFB31
Resection of colon transversum	JGB40, JFB41
Left-sided colectomy	JFB43, JFB44
Resection of sigmoid colon	JFB46, JFB47
Other colon resection	JFB50, JFB51
Proctocolectomy with IPAA (ileal pouch anal anastomosis)	
Colectomy, rectal mucosectomy and ileoanal anastomosis without ileostomy	JFH30
Colectomy, rectal mucosectomy and ileoanal anastomosis and ileostomy	JFH33
Mucosectomy and ileoanal anastomosis after previous colectomy	JGB50
Exstirpation of rectum or making of an ileoanal anastomosis after previous colectomy	JGB60
Continent ileostomy at time of colectomy	
Proctocolectomy with continent ileostomy, Kock	JFH40
Converting a conventional ileostomy to a continent ileostomy	JFG60
Proctocolectomy	
Proctocolektomy with ileostomy	JFH20
**Other bowel surgery**	
Strictureplasty to the small bowel	JFA60
Stricturoplasty to the colon	JFA63
Closure of fistula of small intestine	JFA76
Closure of fistula of colon	JFA86
Colonic and/or small bowel resection	JFB
Formation of stoma	JFF
Operations on intestinal stoma or reservoir	JFG
Other operation of small bowel and/or colon	JFW96
Other laparoscopic operation of small bowel and/or colon	JFW97
Rectal resection	JGB
**Perianal surgery**	
Perianal incision and drainage	JHA00
Dilatation of the anal sphincter	JHD00
Lay open or excision of perianal fistula	JHD20
Partial lay open or excision of perianal fistula (including seton placement)	JHD30

Completion lay open or excision of perianal fistula	JHD33
Excision of perianal fistula with advancement flap	JHD50
Occlusion of perianal fistula with collagen plug	JHD60
Occlusion of perianal fistula with fibrin glue	JHD63
Other anal or perianal surgery (eg, examination under anesthesia)	JHW96

Table S10. Flowchart of identified patients and comparators

	Incident cohort 2007-2021		Prevalent cohort Jul 1st 2008		Overall	
	**Incident UC**	**Population comparators**	**Prevalent UC**	**Population comparators**	**UC**	**Population comparators**
**N individuals**	40 778	403 919	32 423	288 692	73 201	692 611
Excluded due to history of:						
HIV	5	79	10	38	15	117
Chronic hepatitis	332	2 243	378	1 442	710	3685
Transplantation	147	720	222	550	369	1270
Kidney disease	269	1 493	97	494	366	1987
Liver disease	151	475	113	255	264	730
Use before first diagnostic listing of UC or Jul 1st 2008					0	0
Immunomodulators	2 335	4 047	4 829	2 318	7164	6365
TNFi	257	131	116	46	373	177
Vedolizumab	9	0	1	0	10	0
Ustekinumab	2	4	0	0	2	4
Tofacitinib	1	0	0	0	1	0
No matched case or comparator	0	33 333	2	51 871	2	85204
**N included in study**	**37 270**	**361 394**	**26 655**	**231 678**	**63 925**	**593 072**

Table S11. Characteristics of incident patients with UC from January 1st, 2007 to December 31st, 2021, and prevalent patients with UC as of July 1st 2008 and matched population comparators

Characteristic	Incident UC	Population comparators	Prevalent UC	Population comparators
Total	37 270	361 394	26 655	231 678
Sex, n (%)				
Female	18 385 (49.3%)	178 046 (49.3%)	12 594 (47.2%)	110 624 (47.7%)
Male	18 885 (50.7%)	183 348 (50.7%)	14 061 (52.8%)	121 054 (52.3%)
Age at diagnosis (incident patients)/matching (prevalent patients)				
Mean (SD)	44.4 (20.7)	44.1 (20.7)	40.3 (17.1)	39.4 (16.7)
Median (IQR)	40.9 (27.0-61.7)	40.4 (26.8-61.2)	38.4 (27.2-52.4)	37.6 (26.7-51.2)
Categories, n (%)				
<18y	2 773 (7.4%)	27 556 (7.6%)	2 217 (8.3%)	20 519 (8.9%)
18-<40y	15 360 (41.2%)	151 075 (41.8%)	11 962 (44.9%)	106 981 (46.2%)
40-<60y	9 046 (24.3%)	87 554 (24.2%)	8 624 (32.4%)	74 500 (32.2%)
≥60y	10 091 (27.1%)	95 209 (26.3%)	3 852 (14.5%)	29 678 (12.8%)
Age in July 1st, 2008				
Mean (SD)			51.7 (17.0)	50.4 (16.5)
Median (IQR)			51.9 (39.1-63.7)	50.6 (38.2-62.4)
Categories, n (%)				
<18y			406 (1.5%)	3 962 (1.7%)
18-<40y			6 672 (25.0%)	61 481 (26.5%)
40-<60y			10 775 (40.4%)	96 603 (41.7%)
≥60y			8 802 (33.0%)	69 632 (30.1%)
Disease duration in July 1st, 2008				
Mean (SD)			11.4 (9.0)	11.0 (8.8)
Median (IQR)			8.1 (5.1-16.6)	7.6 (4.8-15.7)
Range, min-max			0.0-46.4	0.0-46.4
Categories, n (%)				
0-<1y			1 043 (3.9%)	9 834 (4.2%)
1-<5y			5 525 (20.7%)	50 508 (21.8%)
5-<10y			8 929 (33.5%)	78 756 (34.0%)
≥10y			11 158 (41.9%)	92 580 (40.0%)
Year of diagnosis/matching, n (%)				
2007-2009	7 195 (19.3%)	70 412 (19.5%)		
2010-2012	7 866 (21.1%)	76 582 (21.2%)		
2013-2015	7 692 (20.6%)	74 496 (20.6%)		
2016-2021	14 517 (39.0%)	139 904 (38.7%)		

Education level (years), n (%)				
<9	8 349 (22.4%)	81 314 (22.5%)	6 195 (23.2%)	52 876 (22.8%)
10-12	16 520 (44.3%)	153 686 (42.5%)	12 267 (46.0%)	106 620 (46.0%)
>12	12 149 (32.6%)	122 967 (34.0%)	8 141 (30.5%)	71 579 (30.9%)
Missing	252 (0.7%)	3 427 (0.9%)	52 (0.2%)	603 (0.3%)
Country of birth				
Nordic	32 750 (87.9%)	306 878 (84.9%)	25 238 (94.7%)	214 190 (92.5%)
Non-Nordic	4 517 (12.1%)	54 490 (15.1%)	1 417 (5.3%)	17 485 (7.5%)
Missing	3 (0.0%)	26 (0.0%)	(0.0%)	3 (0.0%)
Co-morbidity past 5 years, n (%)				
Diabetes mellitus[1]	1 631 (6.1%)	10 282 (4.4%)	1 631 (6.1%)	10 282 (4.4%)
Ischemic heart disease[2]	799 (3.0%)	4 719 (2.0%)	799 (3.0%)	4 719 (2.0%)
Hypertension[3]	6 754 (25.3%)	50 950 (22.0%)	6 754 (25.3%)	50 950 (22.0%)
Chronic obstructive pulmonary disease[4]	136 (0.5%)	593 (0.3%)	136 (0.5%)	593 (0.3%)
Cerebrovascular disease[5]	368 (1.4%)	2 144 (0.9%)	368 (1.4%)	2 144 (0.9%)
Rheumatic diseases[6]	180 (0.7%)	643 (0.3%)	180 (0.7%)	643 (0.3%)
Depression and anxiety[7]	4 681 (17.6%)	32 126 (13.9%)	4 681 (17.6%)	32 126 (13.9%)
Medication past 5 years, n (%)				
Drugs against peptic ulcer and reflux	6 249 (23.4%)	32 831 (14.2%)	6 249 (23.4%)	32 831 (14.2%)
Laxatives and bulk forming agents	11 398 (42.8%)	19 197 (8.3%)	11 398 (42.8%)	19 197 (8.3%)
Antidiabetics	1 658 (6.2%)	10 562 (4.6%)	1 658 (6.2%)	10 562 (4.6%)
Non-steroid anti-inflammatory drugs (NSAID)	7 755 (29.1%)	73 426 (31.7%)	7 755 (29.1%)	73 426 (31.7%)
Opioids	6 780 (25.4%)	39 131 (16.9%)	6 780 (25.4%)	39 131 (16.9%)
Antihypertensives	7 310 (27.4%)	55 170 (23.8%)	7 310 (27.4%)	55 170 (23.8%)
Lipid reducers	3 494 (13.1%)	26 410 (11.4%)	3 494 (13.1%)	26 410 (11.4%)
Antibiotics	14 994 (56.3%)	110 074 (47.5%)	14 994 (56.3%)	110 074 (47.5%)
Anticoagulants	4 321 (16.2%)	29 579 (12.8%)	4 321 (16.2%)	29 579 (12.8%)
Drugs for obstructive airway diseases	3 761 (14.1%)	24 079 (10.4%)	3 761 (14.1%)	24 079 (10.4%)
Antidepressants	4 288 (16.1%)	30 559 (13.2%)	4 288 (16.1%)	30 559 (13.2%)
Anxiolytics	3 333 (12.5%)	21 908 (9.5%)	3 333 (12.5%)	21 908 (9.5%)
Hypnotics, sedatives	4 671 (17.5%)	29 624 (12.8%)	4 671 (17.5%)	29 624 (12.8%)
Montreal stage at diagnosis/ July 1st, 2008				

[1] ≥2 main diagnoses of diabetes mellitus in the National Patient Register or ≥2 dispensings of antidiabetic medication in the Prescribed Drug Register

[2] Hospitalization or ≥2 outpatient visits with main diagnosis of ischemic heart disease from a Cardiology or Internal medicine clinic

[3] ≥2 dispensings of antihypertensive medication in the Prescribed Drug Register

[4] ≥2 diagnoses in the National Patient Register

[5] Hospitalization or ≥2 outpatient visits with main diagnosis of cerebrovascular disease from a neurology, stroke or internal medicine clinic

[6] ≥2 diagnoses in the National Patient Register

[7] Hospitalization or ≥2 outpatient visits with main diagnosis of anxiety or depression in the National Patient Register or ≥2 dispensings of antidepressants or anxiolytics in the Prescribed Drug Register

E1 (ulcerative proctitis)	3 284 (12.3%)		3 284 (12.3%)	
E2 (left sided UC)	4 874 (18.3%)		4 874 (18.3%)	
E3 (extensive UC)	11 021 (41.3%)		11 021 (41.3%)	
EX (extent not defined)	6 261 (23.5%)		6 261 (23.5%)	
Missing	1 215 (4.6%)		1 215 (4.6%)	
PCS, n (%)				
At diagnosis	796 (3.0%)	140 (0.1%)	796 (3.0%)	140 (0.1%)
At end of follow-up	1 425 (5.3%)	775 (0.3%)	1 425 (5.3%)	775 (0.3%)
Treatment before first diagnostic listing of UC/ July 1st, 2008, n (%)				
5-ASA	16 557 (62.1%)	455 (0.2%)	16 557 (62.1%)	455 (0.2%)
Colectomy	2 390 (9.0%)	724 (0.3%)	2 390 (9.0%)	724 (0.3%)
Treatment during follow-up, n (%)				
5-ASA	17 469 (65.5%)	2 275 (1.0%)	17 469 (65.5%)	2 275 (1.0%)
Thiopurines	3 040 (11.4%)	821 (0.4%)	3 040 (11.4%)	821 (0.4%)
TNFi	1 731 (6.5%)	1 200 (0.5%)	1 731 (6.5%)	1 200 (0.5%)
Thiopurines + TNFi	503 (1.9%)	101 (0.0%)	503 (1.9%)	101 (0.0%)
Vedolizumab	434 (1.6%)	44 (0.0%)	434 (1.6%)	44 (0.0%)
Ustekinumab	122 (0.5%)	47 (0.0%)	122 (0.5%)	47 (0.0%)
Tofacitinib	54 (0.2%)	28 (0.0%)	54 (0.2%)	28 (0.0%)
Colectomy	988 (3.7%)	2 251 (1.0%)	988 (3.7%)	2 251 (1.0%)
Follow-up time (years)				
Mean (SD)	12.9 (3.6)	13.3 (3.1)	12.9 (3.6)	13.3 (3.1)
Median (IQR)	14.5 (14.5-14.5)	14.5 (14.5-14.5)	14.5 (14.5-14.5)	14.5 (14.5-14.5)
Range, min-max	0.0-14.5	0.0-14.5	0.0-14.5	0.0-14.5
Reason for censoring, n (%)				
Death	5 245 (19.7%)	35 980 (15.5%)	5 245 (19.7%)	35 980 (15.5%)
Emigration	366 (1.4%)	4 454 (1.9%)	366 (1.4%)	4 454 (1.9%)
End of follow-up (Dec 31, 2022)	20 434 (76.7%)	191 125 (82.5%)	20 434 (76.7%)	191 125 (82.5%)
Excluded in analysis of: N (%)				
Colorectal cancer	332 (1.2%)	1 166 (0.5%)	332 (1.2%)	1 166 (0.5%)
Small bowel cancer	15 (0.1%)	55 (0.0%)	15 (0.1%)	55 (0.0%)
Pancreatic cancer	8 (0.0%)	18 (0.0%)	8 (0.0%)	18 (0.0%)
Hepatobiliary cancer	23 (0.1%)	25 (0.0%)	23 (0.1%)	25 (0.0%)
Malignant melanoma	137 (0.5%)	1 006 (0.4%)	137 (0.5%)	1 006 (0.4%)
Lymphoma	67 (0.3%)	624 (0.3%)	67 (0.3%)	624 (0.3%)
Other hematological malignancy	24 (0.1%)	190 (0.1%)	24 (0.1%)	190 (0.1%)
Squamous cell carcinoma of the skin	78 (0.3%)	458 (0.2%)	78 (0.3%)	458 (0.2%)
Basal cell carcinoma	451 (1.7%)	2 797 (1.2%)	451 (1.7%)	2 797 (1.2%)
Cervical cancer (women)	29 (0.1%)	292 (0.1%)	29 (0.1%)	292 (0.1%)
Urinary tract cancer	147 (0.6%)	805 (0.3%)	147 (0.6%)	805 (0.3%)

Breast cancer (women)	400 (1.5%)	2 625 (1.1%)	400 (1.5%)	2 625 (1.1%)
Prostate cancer (men)	347 (1.3%)	2 223 (1.0%)	347 (1.3%)	2 223 (1.0%)
Lung cancer	31 (0.1%)	238 (0.1%)	31 (0.1%)	238 (0.1%)
Uterus (women)	76 (0.3%)	525 (0.2%)	76 (0.3%)	525 (0.2%)
Brain and spinal chord tumors	61 (0.2%)	520 (0.2%)	61 (0.2%)	520 (0.2%)
Other	300 (1.1%)	2 270 (1.0%)	300 (1.1%)	2 270 (1.0%)
All cancer	2 250 (8.4%)	14 298 (6.2%)	2 250 (8.4%)	14 298 (6.2%)

Table S12a. Baseline characteristics of patients with UC and matched population comparators at start of follow-up in naïve patients and patients treated with THFi and/or thiopurine

Characteristic	Naïve		Thiopurines		TNFi		Thiopurines+TNFi	
	UC	Pop comp	UC	Pop comp	UC	Pop comp	UC	Pop comp
Total	54 870	499 761	12 451	116 851	7 507	70 096	3 554	33 557
Sex, n (%)								
Female	27 102 (49.4%)	248 504 (49.7%)	5 402 (43.4%)	50 677 (43.4%)	3 387 (45.1%)	31 588 (45.1%)	1 537 (43.2%)	14 484 (43.2%)
Male	27 768 (50.6%)	251 257 (50.3%)	7 049 (56.6%)	66 174 (56.6%)	4 120 (54.9%)	38 508 (54.9%)	2 017 (56.8%)	19 073 (56.8%)
Age at start of follow-up								
Mean (SD)	42.9 (18.9)	42.3 (18.7)	33.9 (16.8)	33.6 (16.7)	32.2 (15.0)	31.9 (14.9)	30.4 (14.7)	30.2 (14.6)
Median (IQR)	40.2 (27.8-57.1)	39.6 (27.4-56.3)	29.8 (20.9-44.7)	29.5 (20.7-44.1)	28.9 (21.0-41.2)	28.7 (20.8-40.8)	27.1 (19.5-39.3)	26.9 (19.3-38.9)
Categories, n (%)								
<18y	3 746 (6.8%)	35 575 (7.1%)	2 046 (16.4%)	19 809 (17.0%)	1 161 (15.5%)	11 211 (16.0%)	708 (19.9%)	6 883 (20.5%)
18-<40y	23 436 (42.7%)	218 265 (43.7%)	6 500 (52.2%)	61 280 (52.4%)	4 343 (57.9%)	40 564 (57.9%)	2 002 (56.3%)	18 837 (56.1%)
40-<60y	15 990 (29.1%)	144 706 (29.0%)	2 655 (21.3%)	24 649 (21.1%)	1 555 (20.7%)	14 369 (20.5%)	668 (18.8%)	6 278 (18.7%)
≥60y	11 698 (21.3%)	101 215 (20.3%)	1 250 (10.0%)	11 113 (9.5%)	448 (6.0%)	3 952 (5.6%)	176 (5.0%)	1 559 (4.6%)
Disease duration at start of follow-up								
Mean (SD)	6.5 (8.3)	6.1 (7.9)	5.3 (6.5)	5.0 (6.2)	6.6 (7.0)	6.2 (6.7)	5.5 (6.0)	5.3 (5.8)
Median (IQR)	2.0 (1.0-8.5)	1.7 (1.0-8.2)	2.4 (1.4-6.5)	2.3 (1.4-6.0)	3.7 (1.7-8.9)	3.5 (1.7-8.4)	3.1 (1.7-7.1)	3.0 (1.7-6.7)
Range, min-max	1.0-47.4	1.0-47.4	1.0-48.2	1.0-48.2	1.0-49.5	1.0-49.5	1.0-45.1	1.0-45.1
Categories, n (%)								
0-<1y	8 863 (16.2%)	84 227 (16.9%)	83 (0.7%)	801 (0.7%)	21 (0.3%)	203 (0.3%)	8 (0.2%)	79 (0.2%)
1-<5y	24 691 (45.0%)	234 218 (46.9%)	8 612 (69.2%)	82 824 (70.9%)	4 383 (58.4%)	42 158 (60.1%)	2 299 (64.7%)	22 173 (66.1%)
5-<10y	9 301 (17.0%)	81 756 (16.4%)	1 770 (14.2%)	16 143 (13.8%)	1 466 (19.5%)	13 615 (19.4%)	687 (19.3%)	6 430 (19.2%)
≥10y	12 015 (21.9%)	99 560 (19.9%)	1 986 (16.0%)	17 083 (14.6%)	1 637 (21.8%)	14 120 (20.1%)	560 (15.8%)	4 875 (14.5%)
Year at start of follow-up, n (%)								
2008-2011	33 414 (60.9%)	295 677 (59.2%)	2 833 (22.8%)	26 639 (22.8%)	655 (8.7%)	6 151 (8.8%)	366 (10.3%)	3 465 (10.3%)
2012-2014	6 537 (11.9%)	62 473 (12.5%)	2 700 (21.7%)	25 243 (21.6%)	1 120 (14.9%)	10 491 (15.0%)	579 (16.3%)	5 472 (16.3%)
2015-2017	5 838 (10.6%)	55 508 (11.1%)	2 668 (21.4%)	24 975 (21.4%)	1 645 (21.9%)	15 396 (22.0%)	902 (25.4%)	8 550 (25.5%)
2018-2022	9 081 (16.6%)	86 103 (17.2%)	4 250 (34.1%)	39 994 (34.2%)	4 087 (54.4%)	38 058 (54.3%)	1 707 (48.0%)	16 070 (47.9%)
Education level (years), n (%)								
<9	11 939 (21.8%)	107 157 (21.4%)	2 408 (19.3%)	23 721 (20.3%)	1 249 (16.6%)	12 187 (17.4%)	641 (18.0%)	6 366 (19.0%)
10-12	24 802 (45.2%)	222 006 (44.4%)	5 708 (45.8%)	51 487 (44.1%)	3 516 (46.8%)	31 442 (44.9%)	1 626 (45.8%)	14 852 (44.3%)
>12	17 924 (32.7%)	167 780 (33.6%)	4 295 (34.5%)	41 108 (35.2%)	2 724 (36.3%)	26 142 (37.3%)	1 277 (35.9%)	12 197 (36.3%)
Missing	205 (0.4%)	2 818 (0.6%)	40 (0.3%)	535 (0.5%)	18 (0.2%)	325 (0.5%)	10 (0.3%)	142 (0.4%)
Country of birth								
Nordic	49 972 (91.1%)	441 566 (88.4%)	11 048 (88.7%)	101 004 (86.4%)	6 595 (87.9%)	59 639 (85.1%)	3 099 (87.2%)	28 681 (85.5%)
Non-Nordic	4 896 (8.9%)	58 177 (11.6%)	1 402 (11.3%)	15 838 (13.6%)	912 (12.1%)	10 452 (14.9%)	455 (12.8%)	4 874 (14.5%)
Missing	2 (0.0%)	18 (0.0%)	1 (0.0%)	9 (0.0%)	(0.0%)	5 (0.0%)	(0.0%)	2 (0.0%)
Co-morbidity past 5 years, n (%)								

Diabetes mellitus[8]	3 396 (6.2%)	24 235 (4.8%)	593 (4.8%)	3 978 (3.4%)	321 (4.3%)	2 310 (3.3%)	138 (3.9%)	947 (2.8%)
Ischemic heart disease[9]	1 549 (2.8%)	8 668 (1.7%)	177 (1.4%)	982 (0.8%)	75 (1.0%)	451 (0.6%)	22 (0.6%)	182 (0.5%)
Hypertension[10]	14 470 (26.4%)	112 395 (22.5%)	1 971 (15.8%)	15 207 (13.0%)	1 099 (14.6%)	8 160 (11.6%)	419 (11.8%)	3 115 (9.3%)
Chronic obstructive pulmonary disease[11]	323 (0.6%)	1 240 (0.2%)	44 (0.4%)	144 (0.1%)	16 (0.2%)	64 (0.1%)	6 (0.2%)	25 (0.1%)
Cerebrovascular disease[12]	738 (1.3%)	4 513 (0.9%)	74 (0.6%)	518 (0.4%)	27 (0.4%)	226 (0.3%)	12 (0.3%)	88 (0.3%)
Rheumatic diseases[13]	327 (0.6%)	1 303 (0.3%)	81 (0.7%)	302 (0.3%)	258 (3.4%)	176 (0.3%)	42 (1.2%)	77 (0.2%)
Depression and anxiety[14]	11 506 (21.0%)	80 007 (16.0%)	2 261 (18.2%)	16 678 (14.3%)	1 630 (21.7%)	10 897 (15.5%)	693 (19.5%)	4 850 (14.5%)
Medication past 5 years, n (%)								
Drugs against peptic ulcer and reflux	17 296 (31.5%)	86 505 (17.3%)	5 731 (46.0%)	16 702 (14.3%)	3 598 (47.9%)	10 179 (14.5%)	1 723 (48.5%)	4 409 (13.1%)
Laxatives and bulk forming agents	35 297 (64.3%)	58 923 (11.8%)	9 881 (79.4%)	11 496 (9.8%)	5 989 (79.8%)	7 140 (10.2%)	2 841 (79.9%)	3 166 (9.4%)
Antidiabetics	3 532 (6.4%)	25 088 (5.0%)	650 (5.2%)	4 160 (3.6%)	359 (4.8%)	2 408 (3.4%)	149 (4.2%)	985 (2.9%)
Non-steroid anti-inflammatory drugs (NSAID)	19 520 (35.6%)	175 412 (35.1%)	3 516 (28.2%)	35 245 (30.2%)	2 205 (29.4%)	20 748 (29.6%)	924 (26.0%)	9 316 (27.8%)
Opioids	16 940 (30.9%)	102 982 (20.6%)	3 962 (31.8%)	20 326 (17.4%)	2 887 (38.5%)	12 128 (17.3%)	1 230 (34.6%)	5 377 (16.0%)
Antihypertensives	15 876 (28.9%)	122 428 (24.5%)	2 299 (18.5%)	17 325 (14.8%)	1 358 (18.1%)	9 518 (13.6%)	515 (14.5%)	3 728 (11.1%)
Lipid reducers	8 279 (15.1%)	62 256 (12.5%)	1 231 (9.9%)	8 361 (7.2%)	685 (9.1%)	4 293 (6.1%)	283 (8.0%)	1 587 (4.7%)
Antibiotics	35 188 (64.1%)	269 729 (54.0%)	8 160 (65.5%)	59 724 (51.1%)	5 063 (67.4%)	34 480 (49.2%)	2 388 (67.2%)	16 368 (48.8%)
Anticoagulants	10 188 (18.6%)	69 657 (13.9%)	1 588 (12.8%)	9 235 (7.9%)	1 033 (13.8%)	4 977 (7.1%)	404 (11.4%)	1 934 (5.8%)
Drugs for obstructive airway diseases	9 520 (17.4%)	63 978 (12.8%)	2 193 (17.6%)	15 283 (13.1%)	1 362 (18.1%)	9 283 (13.2%)	633 (17.8%)	4 352 (13.0%)
Antidepressants	10 825 (19.7%)	76 367 (15.3%)	2 241 (18.0%)	16 402 (14.0%)	1 584 (21.1%)	10 780 (15.4%)	694 (19.5%)	4 833 (14.4%)
Anxiolytics	8 623 (15.7%)	58 690 (11.7%)	1 773 (14.2%)	12 348 (10.6%)	1 284 (17.1%)	7 864 (11.2%)	534 (15.0%)	3 528 (10.5%)
Hypnotics, sedatives	10 925 (19.9%)	71 966 (14.4%)	2 488 (20.0%)	13 246 (11.3%)	1 751 (23.3%)	8 142 (11.6%)	763 (21.5%)	3 539 (10.5%)
Montreal stage at start of follow-up, n (%)								
E1 (ulcerative proctitis)	11 020 (20.1%)		790 (6.3%)		465 (6.2%)		173 (4.9%)	
E2 (left sided UC)	11 945 (21.8%)		2 906 (23.3%)		1 651 (22.0%)		729 (20.5%)	
E3 (extensive UC)	16 821 (30.7%)		6 424 (51.6%)		4 248 (56.6%)		2 091 (58.8%)	
EX (extent not defined)	13 964 (25.4%)		2 326 (18.7%)		1 135 (15.1%)		561 (15.8%)	
Missing	1 120 (2.0%)		5 (0.0%)		8 (0.1%)		(0.0%)	
PCS, n (%)								
At *start of follow-up*	1 243 (2.3%)	356 (0.1%)	406 (3.3%)	45 (0.0%)	252 (3.4%)	32 (0.0%)	129 (3.6%)	15 (0.0%)
At end of follow-up	2 098 (3.8%)	1 303 (0.3%)	627 (5.0%)	146 (0.1%)	358 (4.8%)	75 (0.1%)	190 (5.3%)	38 (0.1%)
Treatment before start of follow-up, n (%)								
5-ASA	40 600 (74.0%)	1 294 (0.3%)	12 121 (97.3%)	385 (0.3%)	7 228 (96.3%)	312 (0.4%)	3 452 (97.1%)	121 (0.4%)

[8] ≥2 main diagnoses of diabetes mellitus in the National Patient Register or ≥2 dispensings of antidiabetic medication in the Prescribed Drug Register

[9] Hospitalization or ≥2 outpatient visits with main diagnosis of ischemic heart disease from a Cardiology or Internal medicine clinic

[10] ≥2 dispensings of antihypertensive medication in the Prescribed Drug Register

[11] ≥2 diagnoses in the National Patient Register

[12] Hospitalization or ≥2 outpatient visits with main diagnosis of cerebrovascular disease from a neurology, stroke or internal medicine clinic

[13] ≥2 diagnoses in the National Patient Register

[14] Hospitalization or ≥2 outpatient visits with main diagnosis of anxiety or depression in the National Patient Register or ≥2 dispensings of antidepressants or anxiolytics in the Prescribed Drug Register

Colectomy	3 254 (5.9%)	1 899 (0.4%)	694 (5.6%)	252 (0.2%)	742 (9.9%)	143 (0.2%)	316 (8.9%)	54 (0.2%)
Treatment during follow-up, n (%)								
Colectomy	1 534 (2.8%)	3 272 (0.7%)	1 027 (8.2%)	286 (0.2%)	641 (8.5%)	99 (0.1%)	390 (11.0%)	45 (0.1%)
Follow-up time (years)								
Mean (SD)	9.4 (4.6)	9.5 (4.5)	6.9 (4.0)	6.9 (4.0)	5.0 (3.7)	5.0 (3.7)	5.5 (3.6)	5.5 (3.6)
Median (IQR)	11.2 (5.5-13.5)	11.3 (5.7-13.5)	6.9 (3.5-10.4)	6.8 (3.4-10.4)	4.3 (1.9-7.7)	4.3 (1.9-7.6)	5.1 (2.3-8.1)	5.1 (2.3-8.1)
Range, min-max	0.0-15.0	0.0-15.0	0.0-15.0	0.0-15.0	0.0-14.8	0.0-14.8	0.0-14.7	0.0-14.7
Reason for censoring, n (%)								
Death	7 695 (14.0%)	53 539 (10.7%)	501 (4.0%)	3 755 (3.2%)	147 (2.0%)	1 065 (1.5%)	69 (1.9%)	436 (1.3%)
Emigration	775 (1.4%)	9 948 (2.0%)	151 (1.2%)	2 408 (2.1%)	64 (0.9%)	1 161 (1.7%)	32 (0.9%)	642 (1.9%)
End of follow-up (Dec 31, 2022)	46 400 (84.6%)	436 274 (87.3%)	11 798 (94.8%)	110 688 (94.7%)	7 296 (97.2%)	67 870 (96.8%)	3 453 (97.2%)	32 479 (96.8%)
Excluded in analysis of:<br>*N (%)*								
Colorectal cancer	706 (1.3%)	2 727 (0.5%)	47 (0.4%)	316 (0.3%)	25 (0.3%)	156 (0.2%)	8 (0.2%)	54 (0.2%)
Small bowel cancer	29 (0.1%)	114 (0.0%)	2 (0.0%)	11 (0.0%)	1 (0.0%)	12 (0.0%)	(0.0%)	6 (0.0%)
Pancreatic cancer	15 (0.0%)	57 (0.0%)	1 (0.0%)	9 (0.0%)	(0.0%)	7 (0.0%)	(0.0%)	1 (0.0%)
Hepatobiliary cancer	42 (0.1%)	72 (0.0%)	1 (0.0%)	15 (0.0%)	3 (0.0%)	6 (0.0%)	1 (0.0%)	2 (0.0%)
Malignant melanoma	288 (0.5%)	2 263 (0.5%)	34 (0.3%)	334 (0.3%)	21 (0.3%)	188 (0.3%)	3 (0.1%)	80 (0.2%)
Lymphoma	185 (0.3%)	1 370 (0.3%)	16 (0.1%)	204 (0.2%)	10 (0.1%)	101 (0.1%)	4 (0.1%)	37 (0.1%)
Other hematological malignancy	74 (0.1%)	478 (0.1%)	5 (0.0%)	81 (0.1%)	5 (0.1%)	47 (0.1%)	1 (0.0%)	20 (0.1%)
Squamous cell carcinoma of the skin	198 (0.4%)	1 368 (0.3%)	18 (0.1%)	129 (0.1%)	13 (0.2%)	54 (0.1%)	3 (0.1%)	13 (0.0%)
Basal cell carcinoma	1 252 (2.3%)	9 012 (1.8%)	154 (1.2%)	1 267 (1.1%)	113 (1.5%)	722 (1.0%)	37 (1.0%)	255 (0.8%)
Cervical cancer (women)	80 (0.1%)	633 (0.1%)	14 (0.1%)	91 (0.1%)	10 (0.1%)	53 (0.1%)	4 (0.1%)	19 (0.1%)
Urinary tract cancer	335 (0.6%)	1 851 (0.4%)	39 (0.3%)	233 (0.2%)	11 (0.1%)	105 (0.1%)	5 (0.1%)	41 (0.1%)
Breast cancer (women)	821 (1.5%)	5 985 (1.2%)	85 (0.7%)	661 (0.6%)	35 (0.5%)	390 (0.6%)	16 (0.5%)	121 (0.4%)
Prostate cancer (men)	843 (1.5%)	5 395 (1.1%)	79 (0.6%)	687 (0.6%)	27 (0.4%)	314 (0.4%)	6 (0.2%)	110 (0.3%)
Lung cancer	111 (0.2%)	557 (0.1%)	12 (0.1%)	63 (0.1%)	1 (0.0%)	28 (0.0%)	1 (0.0%)	9 (0.0%)
Uterus (women)	172 (0.3%)	1 179 (0.2%)	5 (0.0%)	114 (0.1%)	6 (0.1%)	48 (0.1%)	2 (0.1%)	9 (0.0%)
Brain and spinal chord tumors	145 (0.3%)	1 150 (0.2%)	20 (0.2%)	198 (0.2%)	15 (0.2%)	125 (0.2%)	6 (0.2%)	60 (0.2%)
Other	655 (1.2%)	4 947 (1.0%)	79 (0.6%)	800 (0.7%)	46 (0.6%)	458 (0.7%)	15 (0.4%)	197 (0.6%)
All cancer	5 154 (9.4%)	34 484 (6.9%)	535 (4.3%)	4 690 (4.0%)	298 (4.0%)	2 554 (3.6%)	102 (2.9%)	954 (2.8%)

Table S12b. Baseline characteristics of patients with UC and matched population comparators at start of treatment with vedolizumab, Ustekinumab, and Tofacitinib

Characteristic	Vedolizumab		Ustekinumab		Tofacitinib	
	UC	Pop comp	UC	Pop comp	UC	Pop comp
Total	1 768	16 164	696	6 363	430	3 949
Sex, n (%)						
Female	801 (45.3%)	7 308 (45.2%)	324 (46.6%)	2 984 (46.9%)	184 (42.8%)	1 701 (43.1%)
Male	967 (54.7%)	8 856 (54.8%)	372 (53.4%)	3 379 (53.1%)	246 (57.2%)	2 248 (56.9%)
Age at start of follow-up						
Mean (SD)	34.2 (17.2)	33.7 (17.0)	31.4 (15.1)	31.0 (14.9)	31.8 (13.7)	31.5 (13.6)
Median (IQR)	29.3 (20.8-45.4)	28.9 (20.5-44.5)	28.1 (20.4-39.7)	27.9 (20.1-39.3)	28.9 (21.4-40.6)	28.5 (21.1-40.0)
Categories, n (%)						
<18y	271 (15.3%)	2 582 (16.0%)	123 (17.7%)	1 147 (18.0%)	62 (14.4%)	571 (14.5%)
18-<40y	951 (53.8%)	8 733 (54.0%)	401 (57.6%)	3 702 (58.2%)	256 (59.5%)	2 394 (60.6%)
40-<60y	343 (19.4%)	3 098 (19.2%)	129 (18.5%)	1 159 (18.2%)	95 (22.1%)	844 (21.4%)
≥60y	203 (11.5%)	1 751 (10.8%)	43 (6.2%)	355 (5.6%)	17 (4.0%)	140 (3.5%)
Disease duration at start of follow-up						
Mean (SD)	8.5 (7.5)	8.1 (7.1)	10.1 (7.9)	9.7 (7.6)	9.1 (6.9)	8.7 (6.7)
Median (IQR)	6.2 (3.0-11.5)	5.9 (2.9-11.0)	8.2 (4.4-13.1)	7.9 (4.1-12.9)	7.3 (3.6-12.5)	7.1 (3.5-12.1)
Range, min-max	1.0-47.5	1.0-47.5	1.1-45.4	1.1-45.4	1.0-44.8	1.0-44.8
Categories, n (%)						
0-<1y	1 (0.1%)	10 (0.1%)	(0.0%)	(0.0%)	1 (0.2%)	9 (0.2%)
1-<5y	750 (42.4%)	7 160 (44.3%)	213 (30.6%)	2 038 (32.0%)	147 (34.2%)	1 416 (35.9%)
5-<10y	486 (27.5%)	4 464 (27.6%)	196 (28.2%)	1 829 (28.7%)	124 (28.8%)	1 154 (29.2%)
≥10y	531 (30.0%)	4 530 (28.0%)	287 (41.2%)	2 496 (39.2%)	158 (36.7%)	1 370 (34.7%)
Year at start of follow-up, n (%)						
2008-2011	(0.0%)	(0.0%)	1 (0.1%)	10 (0.2%)	(0.0%)	(0.0%)
2012-2014	1 (0.1%)	10 (0.1%)	1 (0.1%)	8 (0.1%)	(0.0%)	(0.0%)
2015-2017	379 (21.4%)	3 490 (21.6%)	21 (3.0%)	191 (3.0%)	(0.0%)	(0.0%)
2018-2022	1 388 (78.5%)	12 664 (78.3%)	673 (96.7%)	6 154 (96.7%)	430 (100.0%)	3 949 (100.0%)
Education level (years), n (%)						
<9	277 (15.7%)	2 628 (16.3%)	112 (16.1%)	903 (14.2%)	49 (11.4%)	536 (13.6%)
10-12	806 (45.6%)	7 233 (44.7%)	343 (49.3%)	2 878 (45.2%)	209 (48.6%)	1 790 (45.3%)
>12	682 (38.6%)	6 225 (38.5%)	240 (34.5%)	2 554 (40.1%)	172 (40.0%)	1 603 (40.6%)
Missing	3 (0.2%)	78 (0.5%)	1 (0.1%)	28 (0.4%)	(0.0%)	20 (0.5%)
Country of birth						
Nordic	1 560 (88.2%)	13 908 (86.0%)	604 (86.8%)	5 399 (84.8%)	391 (90.9%)	3 384 (85.7%)
Non-Nordic	208 (11.8%)	2 254 (13.9%)	92 (13.2%)	964 (15.2%)	39 (9.1%)	565 (14.3%)
Missing	(0.0%)	2 (0.0%)	(0.0%)	(0.0%)	(0.0%)	(0.0%)

Co-morbidity past 5 years, n (%)						
Diabetes mellitus[15]	107 (6.1%)	683 (4.2%)	42 (6.0%)	254 (4.0%)	18 (4.2%)	143 (3.6%)
Ischemic heart disease[16]	24 (1.4%)	132 (0.8%)	6 (0.9%)	43 (0.7%)	3 (0.7%)	31 (0.8%)
Hypertension[17]	396 (22.4%)	2 635 (16.3%)	143 (20.5%)	924 (14.5%)	67 (15.6%)	534 (13.5%)
Chronic obstructive pulmonary disease[18]	7 (0.4%)	19 (0.1%)	2 (0.3%)	6 (0.1%)	1 (0.2%)	5 (0.1%)
Cerebrovascular disease[19]	9 (0.5%)	80 (0.5%)	3 (0.4%)	24 (0.4%)	(0.0%)	3 (0.1%)
Rheumatic diseases[20]	9 (0.5%)	54 (0.3%)	19 (2.7%)	27 (0.4%)	24 (5.6%)	23 (0.6%)
Depression and anxiety[21]	453 (25.6%)	2 812 (17.4%)	213 (30.6%)	1 214 (19.1%)	107 (24.9%)	739 (18.7%)
Medication past 5 years, n (%)						
Drugs against peptic ulcer and reflux	904 (51.1%)	2 672 (16.5%)	361 (51.9%)	1 008 (15.8%)	214 (49.8%)	655 (16.6%)
Laxatives and bulk forming agents	1 511 (85.5%)	2 011 (12.4%)	588 (84.5%)	788 (12.4%)	365 (84.9%)	421 (10.7%)
Antidiabetics	114 (6.4%)	704 (4.4%)	47 (6.8%)	265 (4.2%)	21 (4.9%)	147 (3.7%)
Non-steroid anti-inflammatory drugs (NSAID)	413 (23.4%)	4 846 (30.0%)	163 (23.4%)	1 912 (30.0%)	107 (24.9%)	1 199 (30.4%)
Opioids	754 (42.6%)	3 040 (18.8%)	323 (46.4%)	1 174 (18.5%)	206 (47.9%)	679 (17.2%)
Antihypertensives	460 (26.0%)	2 947 (18.2%)	161 (23.1%)	1 075 (16.9%)	75 (17.4%)	618 (15.6%)
Lipid reducers	248 (14.0%)	1 379 (8.5%)	93 (13.4%)	462 (7.3%)	53 (12.3%)	250 (6.3%)
Antibiotics	1 230 (69.6%)	7 746 (47.9%)	504 (72.4%)	2 963 (46.6%)	282 (65.6%)	1 786 (45.2%)
Anticoagulants	377 (21.3%)	1 633 (10.1%)	166 (23.9%)	566 (8.9%)	84 (19.5%)	302 (7.6%)
Drugs for obstructive airway diseases	374 (21.2%)	2 233 (13.8%)	145 (20.8%)	880 (13.8%)	82 (19.1%)	567 (14.4%)
Antidepressants	448 (25.3%)	2 759 (17.1%)	201 (28.9%)	1 222 (19.2%)	110 (25.6%)	730 (18.5%)
Anxiolytics	325 (18.4%)	1 977 (12.2%)	150 (21.6%)	797 (12.5%)	74 (17.2%)	506 (12.8%)
Hypnotics, sedatives	527 (29.8%)	2 158 (13.4%)	243 (34.9%)	857 (13.5%)	130 (30.2%)	520 (13.2%)
Montreal stage at start of follow-up, n (%)						
E1 (ulcerative proctitis)	64 (3.6%)		24 (3.4%)		10 (2.3%)	
E2 (left sided UC)	373 (21.1%)		138 (19.8%)		92 (21.4%)	
E3 (extensive UC)	1 175 (66.5%)		466 (67.0%)		309 (71.9%)	
EX (extent not defined)	156 (8.8%)		67 (9.6%)		19 (4.4%)	
Missing	(0.0%)		1 (0.1%)		(0.0%)	
PCS, n (%)						
At *start of follow-up*	89 (5.0%)	13 (0.1%)	37 (5.3%)	7 (0.1%)	17 (4.0%)	4 (0.1%)
At end of follow-up	115 (6.5%)	19 (0.1%)	43 (6.2%)	7 (0.1%)	20 (4.7%)	4 (0.1%)
Treatment before start of follow-up, n (%)						

[15] ≥2 main diagnoses of diabetes mellitus in the National Patient Register or ≥2 dispensings of antidiabetic medication in the Prescribed Drug Register

[16] Hospitalization or ≥2 outpatient visits with main diagnosis of ischemic heart disease from a Cardiology or Internal medicine clinic

[17] ≥2 dispensings of antihypertensive medication in the Prescribed Drug Register

[18] ≥2 diagnoses in the National Patient Register

[19] Hospitalization or ≥2 outpatient visits with main diagnosis of cerebrovascular disease from a neurology, stroke or internal medicine clinic

[20] ≥2 diagnoses in the National Patient Register

[21] Hospitalization or ≥2 outpatient visits with main diagnosis of anxiety or depression in the National Patient Register or ≥2 dispensings of antidepressants or anxiolytics in the Prescribed Drug Register

Colectomy	211 (11.9%)	43 (0.3%)	123 (17.7%)	18 (0.3%)	81 (18.8%)	5 (0.1%)
Treatment during follow-up, n (%)						
Colectomy	182 (10.3%)	21 (0.1%)	52 (7.5%)	3 (0.0%)	25 (5.8%)	3 (0.1%)
Follow-up time (years)						
Mean (SD)	8.5 (7.5)	8.1 (7.1)	10.1 (7.9)	9.7 (7.6)	9.1 (6.9)	8.7 (6.7)
Median (IQR)	6.2 (3.0-11.5)	5.9 (2.9-11.0)	8.2 (4.4-13.1)	7.9 (4.1-12.9)	7.3 (3.6-12.5)	7.1 (3.5-12.1)
Range, min-max	1.0-47.5	1.0-47.5	1.1-45.4	1.1-45.4	1.0-44.8	1.0-44.8
Reason for censoring, n (%)						
Death	29 (1.6%)	183 (1.1%)	6 (0.9%)	29 (0.5%)	5 (1.2%)	21 (0.5%)
Emigration	9 (0.5%)	148 (0.9%)	2 (0.3%)	30 (0.5%)	1 (0.2%)	19 (0.5%)
End of follow-up (Dec 31, 2020)	1 730 (97.9%)	15 833 (98.0%)	688 (98.9%)	6 304 (99.1%)	424 (98.6%)	3 909 (99.0%)
Excluded in analysis of:<br>*N (%)*						
Colorectal cancer	14 (0.8%)	56 (0.3%)	4 (0.6%)	19 (0.3%)	1 (0.2%)	8 (0.2%)
Small bowel cancer	1 (0.1%)	1 (0.0%)	(0.0%)	(0.0%)	(0.0%)	(0.0%)
Pancreatic cancer	(0.0%)	2 (0.0%)	(0.0%)	(0.0%)	(0.0%)	(0.0%)
Hepatobiliary cancer	1 (0.1%)	1 (0.0%)	(0.0%)	(0.0%)	(0.0%)	(0.0%)
Malignant melanoma	19 (1.1%)	82 (0.5%)	10 (1.4%)	16 (0.3%)	3 (0.7%)	9 (0.2%)
Lymphoma	2 (0.1%)	34 (0.2%)	1 (0.1%)	12 (0.2%)	(0.0%)	7 (0.2%)
Other hematological malignancy	4 (0.2%)	15 (0.1%)	3 (0.4%)	2 (0.0%)	1 (0.2%)	4 (0.1%)
Squamous cell carcinoma of the skin	12 (0.7%)	33 (0.2%)	2 (0.3%)	10 (0.2%)	1 (0.2%)	1 (0.0%)
Basal cell carcinoma	53 (3.0%)	323 (2.0%)	20 (2.9%)	88 (1.4%)	9 (2.1%)	35 (0.9%)
Cervical cancer (women)	1 (0.1%)	13 (0.1%)	1 (0.1%)	5 (0.1%)	(0.0%)	3 (0.1%)
Urinary tract cancer	6 (0.3%)	37 (0.2%)	1 (0.1%)	6 (0.1%)	(0.0%)	3 (0.1%)
Breast cancer (women)	18 (1.0%)	143 (0.9%)	2 (0.3%)	42 (0.7%)	1 (0.2%)	31 (0.8%)
Prostate cancer (men)	20 (1.1%)	123 (0.8%)	5 (0.7%)	44 (0.7%)	(0.0%)	13 (0.3%)
Lung cancer	3 (0.2%)	17 (0.1%)	(0.0%)	1 (0.0%)	(0.0%)	3 (0.1%)
Uterus (women)	2 (0.1%)	26 (0.2%)	(0.0%)	6 (0.1%)	(0.0%)	3 (0.1%)
Brain and spinal chord tumors	5 (0.3%)	30 (0.2%)	1 (0.1%)	18 (0.3%)	2 (0.5%)	11 (0.3%)
Other	12 (0.7%)	133 (0.8%)	5 (0.7%)	51 (0.8%)	(0.0%)	25 (0.6%)
All cancer	150 (8.5%)	921 (5.7%)	47 (6.8%)	285 (4.5%)	17 (4.0%)	139 (3.5%)

Table S13a. Number, n, and proportion (%) of events of ulcerative colitis (UC)-associated cancers, cancers with known/suspected association with immunomodulatory treatment, and cancers common in the population during follow-up in patients with UC, stratified by treatment at start of follow-up: naive (no treatment with thiopurine, tumor necrosis factor inhibitors (TNFi) and other targeted therapies), thiopurines (treatment with thiopurines), TNFi (treatment with TNFi), and thiopurines+TNFi (overlapping treatment with thiopurine and TNFi)

	Treatment cohort							
	Naïve		Thiopurines		TNFi		Thiopurines +TNFi	
Cancer type	UC N (%) events	Pop comp N (%) events	UC N (%) events	Pop comp N (%) events	UC N (%) events	Pop comp N (%) events	UC N (%) events	Pop comp N (%) events
N	49 688	428 137	11 916	108 049	7 209	65 222	3 452	31 762
Follow-up time (years)								
Mean (SD)	9.6 (4.5)	9.7 (4.5)	7.0 (4.0)	7.0 (4.0)	5.0 (3.7)	5.0 (3.7)	5.5 (3.6)	5.5 (3.7)
Median (IQR)	11.7 (5.9-13.5)	11.8 (6.1-13.5)	6.9 (3.5-10.4)	6.9 (3.4-10.5)	4.3 (1.9-7.7)	4.3 (1.9-7.7)	5.1 (2.3-8.1)	5.1 (2.3-8.1)
**Any cancer**								
N events (%)	5208 (10.5)	39772 (9.3)	718 (6.0)	4091 (3.8)	262 (3.6)	1515 (2.3)	121 (3.5)	704 (2.2)
Cum 5-year incidence	5.3% (5.1-5.5)	4.1% (4.1-4.2)	4.3% (3.9-4.7)	2.5% (2.4-2.6)	3.6% (3.1-4.2)	2.1% (2.0-2.3)	3.4% (2.8-4.2)	1.9% (1.7-2.1)
UC-associated cancer								
Colorectal cancer	515 (1.0)	4580 (0.9)	95 (0.8)	413 (0.4)	35 (0.5)	145 (0.2)	20 (0.6)	59 (0.2)
Small bowel cancer	38 (0.1)	240 (0.0)	3 (0.0)	14 (0.0)	1 (0.0)	2 (0.0)	0 (0.0)	1 (0.0)
Pancreatic cancer	113 (0.2)	940 (0.2)	9 (0.1)	85 (0.1)	4 (0.1)	25 (0.0)	2 (0.1)	11 (0.0)
Hepatobiliary cancer	279 (0.5)	765 (0.2)	34 (0.3)	60 (0.1)	20 (0.3)	28 (0.0)	8 (0.2)	15 (0.0)
Known/suspected association with immunomodulatory medication								
Malignant melanoma	294 (0.5)	2757 (0.6)	35 (0.3)	327 (0.3)	15 (0.2)	122 (0.2)	10 (0.3)	63 (0.2)
Lymphoma	225 (0.4)	1888 (0.4)	33 (0.3)	182 (0.2)	20 (0.3)	65 (0.1)	14 (0.4)	21 (0.1)
Other hematological malignancy	118 (0.2)	1325 (0.3)	19 (0.2)	138 (0.1)	6 (0.1)	47 (0.1)	5 (0.1)	20 (0.1)
Squamous cell carcinoma of the skin	363 (0.7)	3339 (0.7)	96 (0.8)	261 (0.2)	17 (0.2)	95 (0.1)	8 (0.2)	35 (0.1)
Basal cell carcinoma	2094 (3.9)	15942 (3.3)	296 (2.4)	1493 (1.3)	101 (1.4)	561 (0.8)	42 (1.2)	256 (0.8)
Cervical cancer (women)	22 (0.1)	312 (0.1)	4 (0.1)	43 (0.1)	2 (0.1)	19 (0.1)	2 (0.1)	3 (0.0)
Urinary tract cancer	341 (0.6)	3070 (0.6)	25 (0.2)	300 (0.3)	6 (0.1)	100 (0.1)	2 (0.1)	42 (0.1)
Other common cancers:								
Breast cancer (women)	523 (2.0)	4943 (2.1)	50 (0.9)	471 (1.0)	16 (0.5)	186 (0.6)	5 (0.3)	93 (0.7)
Prostate cancer (men)	851 (3.2)	7754 (3.2)	94 (1.3)	767 (1.2)	28 (0.7)	242 (0.6)	12 (0.6)	115 (0.6)

Lung cancer	330 (0.6)	3101 (0.6)	26 (0.2)	258 (0.2)	12 (0.2)	80 (0.1)	4 (0.1)	30 (0.1)
Uterine cancer (women)	98 (0.4)	1079 (0.4)	9 (0.2)	102 (0.2)	4 (0.1)	31 (0.1)	0 (0.0)	18 (0.1)
Brain or spinal chord tumors	113 (0.2)	964 (0.2)	11 (0.1)	114 (0.1)	2 (0.0)	52 (0.1)	2 (0.1)	23 (0.1)
Other	614 (1.1)	5370 (1.1)	87 (0.7)	616 (0.5)	34 (0.5)	242 (0.3)	13 (0.4)	115 (0.3)

pop comp, matched comparators from the general population

Table S13b. Number, n, and proportion (%) of events of ulcerative colitis (UC)-associated cancers, cancers with known/suspected association with immunomodulatory treatment, and cancers common in the population during follow-up in patients with UC, stratified by treatment at start of follow-up: vedolizumab, ustekinumab, and tofacitinib

	Treatment cohort					
	**Vedolizumab**		**Ustekinumab**		**Tofacitinib**	
**Cancer type**	**UC N (%) events**	**Pop comp N (%) events**	**UC N (%) events**	**Pop comp N (%) events**	**UC N (%) events**	**Pop comp N (%) events**
N	1 618	14 171	649	5 718	413	3 674
Follow-up time (years)						
Mean (SD)	3.0 (2.1)	3.1 (2.1)	1.9 (1.5)	1.9 (1.5)	1.7 (1.0)	1.7 (1.0)
Median (IQR)	2.8 (1.1-4.7)	2.8 (1.1-4.8)	1.6 (0.8-2.4)	1.6 (0.8-2.5)	1.7 (0.8-2.6)	1.7 (0.8-2.7)
**Any cancer**						
N events (%)	40 (2.5)	231 (1.6)	7 (1.1)	56 (1.0)	3 (0.7)	31 (0.8)
Cum 5-year incidence	3.9% (2.8-5.6)	2.6% (2.3-3.0)	1.4% (0.7-3.1)	2.4% (1.6-3.6)	1.0% (0.3-3.1)	2.7% (1.1-6.6)
UC-associated cancer						
Colorectal cancer	5 (0.3)	31 (0.2)	0 (0.0)	5 (0.1)	1 (0.2)	5 (0.1)
Small bowel cancer	0 (0.0)	1 (0.0)	0 (0.0)	1 (0.0)	0 (0.0)	0 (0.0)
Pancreatic cancer	1 (0.1)	3 (0.0)	0 (0.0)	1 (0.0)	1 (0.2)	1 (0.0)
Hepatobiliary cancer	1 (0.1)	1 (0.0)	1 (0.1)	0 (0.0)	0 (0.0)	0 (0.0)
Known/suspected association with immunomodulatory medication						
Malignant melanoma	2 (0.1)	25 (0.2)	0 (0.0)	4 (0.1)	1 (0.2)	5 (0.1)
Lymphoma	3 (0.2)	11 (0.1)	1 (0.1)	0 (0.0)	0 (0.0)	2 (0.1)
Other hematological malignancy	0 (0.0)	7 (0.0)	0 (0.0)	4 (0.1)	0 (0.0)	0 (0.0)
Squamous cell carcinoma of the skin	8 (0.5)	20 (0.1)	2 (0.3)	4 (0.1)	0 (0.0)	2 (0.1)
Basal cell carcinoma	16 (0.9)	87 (0.6)	5 (0.7)	17 (0.3)	0 (0.0)	10 (0.3)
Cervical cancer (women)	0 (0.0)	1 (0.0)	0 (0.0)	1 (0.0)	0 (0.0)	0 (0.0)
Urinary tract cancer	2 (0.1)	16 (0.1)	0 (0.0)	3 (0.0)	0 (0.0)	0 (0.0)
Other common cancers:						
Breast cancer (women)	2 (0.3)	43 (0.6)	0 (0.0)	8 (0.3)	0 (0.0)	3 (0.2)
Prostate cancer (men)	6 (0.6)	35 (0.4)	0 (0.0)	3 (0.1)	0 (0.0)	7 (0.3)
Lung cancer	1 (0.1)	14 (0.1)	0 (0.0)	4 (0.1)	0 (0.0)	2 (0.1)

Uterine cancer (women)	0 (0.0)	8 (0.1)	0 (0.0)	0 (0.0)	0 (0.0)	0 (0.0)
Brain or spinal chord tumors	0 (0.0)	5 (0.0)	0 (0.0)	1 (0.0)	0 (0.0)	1 (0.0)
Other	5 (0.3)	34 (0.2)	1 (0.1)	12 (0.2)	2 (0.5)	3 (0.1)

Table S14a. Incidence rate (cases/1000 person years) and IR differences of UC-associated cancers, cancers with known/suspected association with immunomodulatory treatment, and cancers common in the population different cancers up in cohorts of patients with UC, stratified by treatment at start of follow-up: naive (no past or ongoing treatment with thiopurines, tumor necrosis factor inhibitors (TNFi) and other targeted therapies), thiopurine (treatment with thiopurine), TNFi (treatment with TNFi), and thiopurine+TNFi (overlapping treatment with thiopurine and TNFi)

	**Treatment cohort**											
	**Naïve**			**Thiopurines**			**TNFi**			**Thiopurines+TNFi**		
**Cancer type**	**UC IR (95% CI)**	**Pop comp IR (95% CI)**	**IR difference (95% CI)**	**UC IR (95% CI)**	**Pop comp IR (95% CI)**	**IR difference (95% CI)**	**UC IR (95% CI)**	**Pop comp IR (95% CI)**	**IR difference (95% CI)**	**UC IR (95% CI)**	**Pop comp IR (95% CI)**	**IR difference (95% CI)**
Any cancer	12.70 (12.35-13.04)	10.04 (9.94-10.14)	2.66 (2.30-3.01)	8.95 (8.29-9.60)	5.56 (5.39-5.73)	3.38 (2.71-4.06)	7.39 (6.49-8.28)	4.70 (4.46-4.94)	2.69 (1.76-3.61)	6.53 (5.37-7.70)	4.12 (3.81-4.42)	2.42 (1.21-3.62)
Age-sex standardised	11.73 (11.41-12.05)	10.87 (10.76-10.98)		16.31 (15.12-17.50)	9.72 (9.42-10.02)		13.97 (12.28-15.66)	10.78 (10.23-11.32)		9.93 (8.16-11.70)	13.58 (12.57-14.58)	
UC-associated cancer												
Colorectal cancer	1.11 (1.01-1.21)	0.99 (0.96-1.01)	0.12 (0.02-0.22)	1.11 (0.89-1.33)	0.52 (0.47-0.56)	0.59 (0.36-0.82)	0.94 (0.63-1.25)	0.42 (0.35-0.49)	0.52 (0.20-0.84)	1.03 (0.58-1.48)	0.32 (0.24-0.41)	0.71 (0.25-1.17)
Small bowel cancer	0.08 (0.06-0.11)	0.05 (0.04-0.06)	0.03 (0.00-0.06)	0.03 (0.00-0.07)	0.02 (0.01-0.03)	0.02 (-0.02-0.06)	0.03 (0.00-0.08)	0.01 (0.00-0.01)	0.02 (-0.03-0.07)	0.00 (0.00-0.00)	0.01 (0.00-0.02)	
Pancreatic cancer	0.24 (0.20-0.28)	0.20 (0.19-0.21)	0.04 (-0.00-0.09)	0.10 (0.04-0.17)	0.11 (0.08-0.13)	-0.00 (-0.07-0.07)	0.11 (0.00-0.21)	0.07 (0.04-0.10)	0.03 (-0.07-0.14)	0.10 (0.00-0.25)	0.06 (0.02-0.10)	0.04 (-0.10-0.19)
Hepatobiliary cancer	0.59 (0.52-0.66)	0.16 (0.15-0.17)	0.43 (0.36-0.50)	0.39 (0.26-0.53)	0.07 (0.06-0.09)	0.32 (0.19-0.45)	0.53 (0.30-0.77)	0.08 (0.05-0.11)	0.45 (0.22-0.69)	0.41 (0.13-0.70)	0.08 (0.04-0.12)	0.33 (0.04-0.62)
Known/suspected association with immunomodulatory medication												
Malignant melanoma	0.63 (0.56-0.70)	0.59 (0.57-0.61)	0.04 (-0.04-0.11)	0.41 (0.27-0.54)	0.41 (0.36-0.45)	-0.00 (-0.14-0.14)	0.40 (0.20-0.60)	0.35 (0.29-0.41)	0.05 (-0.16-0.26)	0.52 (0.20-0.84)	0.35 (0.26-0.43)	0.17 (-0.16-0.50)
Lymphoma	0.48 (0.42-0.54)	0.40 (0.38-0.42)	0.08 (0.01-0.14)	0.38 (0.25-0.51)	0.23 (0.19-0.26)	0.16 (0.02-0.29)	0.53 (0.30-0.77)	0.19 (0.14-0.23)	0.35 (0.11-0.59)	0.72 (0.34-1.10)	0.12 (0.07-0.16)	0.61 (0.23-0.99)
Other hematological malignancy	0.25 (0.21-0.30)	0.28 (0.27-0.30)	-0.03 (-0.08-0.02)	0.22 (0.12-0.32)	0.17 (0.14-0.20)	0.05 (-0.05-0.15)	0.16 (0.03-0.29)	0.13 (0.10-0.17)	0.03 (-0.11-0.16)	0.26 (0.03-0.48)	0.11 (0.06-0.16)	0.15 (-0.08-0.38)
Squamous cell carcinoma of the skin	0.77 (0.69-0.85)	0.71 (0.69-0.73)	0.06 (-0.02-0.15)	1.12 (0.89-1.34)	0.32 (0.28-0.36)	0.79 (0.57-1.02)	0.45 (0.24-0.67)	0.27 (0.22-0.33)	0.18 (-0.04-0.40)	0.41 (0.13-0.70)	0.19 (0.13-0.26)	0.22 (-0.07-0.51)
Basal cell carcinoma	4.63 (4.43-4.83)	3.54 (3.48-3.59)	1.10 (0.89-1.30)	3.52 (3.12-3.92)	1.90 (1.80-2.00)	1.62 (1.21-2.03)	2.75 (2.21-3.29)	1.65 (1.51-1.79)	1.10 (0.55-1.66)	2.20 (1.53-2.86)	1.43 (1.26-1.61)	0.77 (0.08-1.46)
Cervical cancer (women)	0.09 (0.06-0.13)	0.13 (0.12-0.15)	-0.04 (-0.08-0.00)	0.11 (0.00-0.21)	0.12 (0.09-0.16)	-0.02 (-0.13-0.10)	0.12 (0.00-0.29)	0.12 (0.07-0.18)	-0.00 (-0.18-0.17)	0.24 (0.00-0.57)	0.04 (0.00-0.08)	0.20 (-0.13-0.54)
Urinary tract cancer	0.73 (0.65-0.81)	0.66 (0.63-0.68)	0.07 (-0.01-0.15)	0.29 (0.18-0.40)	0.37 (0.33-0.42)	-0.08 (-0.20-0.04)	0.16 (0.03-0.29)	0.29 (0.23-0.34)	-0.13 (-0.27-0.01)	0.10 (0.00-0.25)	0.23 (0.16-0.30)	-0.13 (-0.29-0.03)
Common cancers:												
Breast cancer (women)	2.34 (2.14-2.54)	2.24 (2.18-2.30)	0.10 (-0.11-0.31)	1.36 (0.98-1.74)	1.38 (1.26-1.51)	-0.03 (-0.42-0.37)	0.97 (0.50-1.45)	1.23 (1.06-1.41)	-0.26 (-0.77-0.25)	0.60 (0.07-1.13)	1.22 (0.97-1.46)	-0.61 (-1.20--0.03)
Prostate cancer (men)	3.73 (3.48-3.98)	3.43 (3.36-3.51)	0.29 (0.03-0.56)	1.97 (1.57-2.36)	1.73 (1.60-1.85)	0.24 (-0.18-0.66)	1.35 (0.85-1.85)	1.26 (1.10-1.42)	0.09 (-0.44-0.62)	1.09 (0.47-1.71)	1.11 (0.91-1.31)	-0.02 (-0.67-0.63)
Lung cancer	0.70 (0.63-0.78)	0.66 (0.63-0.68)	0.04 (-0.03-0.12)	0.30 (0.19-0.42)	0.32 (0.28-0.36)	-0.02 (-0.14-0.10)	0.32 (0.14-0.50)	0.23 (0.18-0.28)	0.09 (-0.10-0.28)	0.21 (0.00-0.41)	0.16 (0.11-0.22)	0.04 (-0.17-0.25)
Uterine (women)	0.42 (0.34-0.51)	0.47 (0.44-0.49)	-0.04 (-0.13-0.05)	0.24 (0.08-0.40)	0.29 (0.24-0.35)	-0.05 (-0.22-0.12)	0.24 (0.00-0.48)	0.20 (0.13-0.27)	0.04 (-0.21-0.29)	0.00 (0.00-0.00)	0.23 (0.12-0.34)	-0.23 (-0.23--0.23)
Brain or spinal chord tumors	0.24 (0.20-0.28)	0.20 (0.19-0.22)	0.04 (-0.01-0.08)	0.13 (0.05-0.20)	0.14 (0.12-0.17)	-0.01 (-0.09-0.07)	0.05 (0.00-0.13)	0.15 (0.11-0.19)	-0.10 (-0.18--0.01)	0.10 (0.00-0.25)	0.13 (0.07-0.18)	-0.02 (-0.18-0.13)
Other	1.32 (1.22-1.43)	1.16 (1.13-1.19)	0.16 (0.05-0.27)	1.02 (0.80-1.23)	0.77 (0.71-0.84)	0.24 (0.02-0.47)	0.91 (0.61-1.22)	0.70 (0.62-0.79)	0.21 (-0.11-0.53)	0.67 (0.31-1.04)	0.64 (0.52-0.75)	0.04 (-0.35-0.42)

IR, incidence rate; pop comp, general population comparators

Table S14b. Incidence rate (cases/1000 person years) and IR differences of UC-associated cancers, cancers with known/suspected association with immunomodulatory treatment, and cancers common in the population different cancers up in cohorts of patients with UC, stratified by treatment at start of follow-up: vedolizumab, Ustekinumab, and tofacitinib

	Treatment cohort								
	Vedolizumab			Ustekinumab			Tofacitinib		
Cancer type	UC IR (95% CI)	Pop comp IR (95% CI)	IR difference (95% CI)	UC IR (95% CI)	Pop comp IR (95% CI)	IR difference (95% CI)	UC IR (95% CI)	Pop comp IR (95% CI)	IR difference (95% CI)
Any cancer	8.27 (5.71-10.83)	5.39 (4.69-6.08)	2.88 (0.23-5.54)	5.87 (1.52-10.22)	5.30 (3.91-6.69)	0.57 (-3.99-5.13)	4.31 (0.00-9.19)	5.00 (3.24-6.76)	-0.69 (-5.88-4.50)
Age-sex standardised	13.43 (9.27-17.59)	9.05 (7.88-10.22)		28.81 (7.47-50.15)	11.53 (8.51-14.54)		3.20 (-0.42-6.83)	6.28 (4.07-8.49)	
UC-associated cancer									
Colorectal cancer	0.96 (0.12-1.80)	0.65 (0.42-0.88)	0.31 (-0.56-1.18)	0.00 (0.00-0.00)	0.44 (0.05-0.82)		1.39 (0.00-4.10)	0.75 (0.09-1.41)	0.63 (-2.16-3.43)
Small bowel cancer	0.00 (0.00-0.00)	0.02 (0.00-0.06)		0.00 (0.00-0.00)	0.09 (0.00-0.26)		0.00 (0.00-0.00)	0.00 (0.00-0.00)	0.00 (0.00-0.00)
Pancreatic cancer	0.19 (0.00-0.56)	0.06 (0.00-0.13)	0.13 (-0.25-0.51)	0.00 (0.00-0.00)	0.09 (0.00-0.26)		1.38 (0.00-4.09)	0.15 (0.00-0.44)	1.23 (-1.49-3.95)
Hepatobiliary cancer	0.19 (0.00-0.56)	0.02 (0.00-0.06)	0.17 (-0.21-0.55)	0.80 (0.00-2.36)	0.00 (0.00-0.00)		0.00 (0.00-0.00)	0.00 (0.00-0.00)	0.00 (0.00-0.00)
Known/suspected association with immunomodulatory medication									
Malignant melanoma	0.38 (0.00-0.92)	0.52 (0.32-0.73)	-0.14 (-0.71-0.43)	0.00 (0.00-0.00)	0.35 (0.01-0.70)		1.39 (0.00-4.10)	0.75 (0.09-1.42)	0.63 (-2.16-3.43)
Lymphoma	0.57 (0.00-1.22)	0.23 (0.09-0.36)	0.34 (-0.32-1.01)	0.80 (0.00-2.36)	0.00 (0.00-0.00)		0.00 (0.00-0.00)	0.30 (0.00-0.72)	
Other hematological malignancy	0.00 (0.00-0.00)	0.15 (0.04-0.25)	-	0.00 (0.00-0.00)	0.35 (0.01-0.69)		0.00 (0.00-0.00)	0.00 (0.00-0.00)	
Squamous cell carcinoma of the skin	1.53 (0.47-2.60)	0.42 (0.23-0.60)	1.12 (0.04-2.20)	1.60 (0.00-3.82)	0.35 (0.01-0.69)	1.25 (-0.99-3.50)	0.00 (0.00-0.00)	0.30 (0.00-0.72)	
Basal cell carcinoma	3.15 (1.61-4.69)	1.89 (1.49-2.28)	1.26 (-0.33-2.85)	4.09 (0.51-7.68)	1.53 (0.80-2.25)	2.56 (-1.10-6.22)	0.00 (0.00-0.00)	1.54 (0.59-2.49)	
Cervical cancer (women)	0.00 (0.00-0.00)	0.05 (0.00-0.14)		0.00 (0.00-0.00)	0.17 (0.00-0.51)		0.00 (0.00-0.00)	0.00 (0.00-0.00)	
Urinary tract cancer	0.38 (0.00-0.91)	0.33 (0.17-0.50)	0.05 (-0.51-0.60)	0.00 (0.00-0.00)	0.26 (0.00-0.56)		0.00 (0.00-0.00)	0.00 (0.00-0.00)	
Common cancers:									
Breast cancer (women)	0.86 (0.00-2.05)	2.06 (1.44-2.68)	-1.20 (-2.54-0.14)	0.00 (0.00-0.00)	1.40 (0.43-2.37)		0.00 (0.00-0.00)	1.11 (0.00-2.37)	
Prostate cancer (men)	2.12 (0.42-3.82)	1.35 (0.91-1.80)	0.77 (-0.99-2.53)	0.00 (0.00-0.00)	0.53 (0.00-1.14)		0.00 (0.00-0.00)	1.81 (0.47-3.15)	
Lung cancer	0.19 (0.00-0.56)	0.29 (0.14-0.44)	-0.10 (-0.50-0.30)	0.00 (0.00-0.00)	0.35 (0.01-0.69)		0.00 (0.00-0.00)	0.30 (0.00-0.72)	
Uterine (women)	0.00 (0.00-0.00)	0.37 (0.11-0.63)		0.00 (0.00-0.00)	0.00 (0.00-0.00)		0.00 (0.00-0.00)	0.00 (0.00-0.00)	
Brain or spinal chord tumors	0.00 (0.00-0.00)	0.10 (0.01-0.20)		0.00 (0.00-0.00)	0.09 (0.00-0.26)		0.00 (0.00-0.00)	0.15 (0.00-0.45)	
Other	0.96 (0.12-1.80)	0.71 (0.47-0.95)	0.24 (-0.63-1.12)	0.80 (0.00-2.36)	1.05 (0.46-1.65)	-0.26 (-1.93-1.42)	2.77 (0.00-6.61)	0.45 (0.00-0.97)	2.32 (-1.56-6.19)

Table S15. Hazard ratios and incidence rate differences, stratified by presence of primary biliary cirrhosis (PSC) at baseline

**Patients with PSC at baseline**	**Treatment cohort**			
**Hazard Ratio**	**Naïve**	**Thiopurines**	**TNFi**	**Thiopurines+TNFi**
**Hepatobiliary cancer**	43.42 (24.92-75.65)			
Age at start of follow-up				
<18y				
18-<40y				
40-<60y	63.44 (22.26-180.84)			
≥60y	9.75 (4.44-21.40)			
**IR Difference**				
**Hepatobiliary cancer**	6.89 (5.31-8.47)			
Age at start of follow-up				
<18y				
18-<40y				
40-<60y	7.74 (4.87-10.61)			
≥60y	5.41 (2.43-8.40)			
**Patients without PSC at baseline**	**Treatment cohort**			
**Hazard Ratio**	**Naïve**	**Thiopurines**	**TNFi**	**Thiopurines+TNFi**
**Hepatobiliary cancer**	2.57 (2.19-3.01)	3.01 (1.78-5.08)	2.91 (1.41-6.03)	1.88 (0.54-6.50)
Age at start of follow-up				
<18y	-	-		

18-<40y	19.59 (10.13-37.88)	6.05 (1.71-21.47)	2.99 (0.31-28.74)	
40-<60y	3.26 (2.41-4.43)	3.00 (1.09-8.26)	4.16 (1.25-13.84)	2.99 (0.31-28.74)
≥60y	1.90 (1.54-2.33)	2.22 (1.06-4.65)	1.83 (0.61-5.50)	1.83 (0.21-15.68)
**IR Difference**				
**Hepatobiliary cancer**	0.28 (0.22-0.34)	0.15 (0.05-0.26)	0.19 (0.02-0.37)	0.07 (-0.11-0.26)
Age at start of follow-up				
<18y	-			
18-<40y	0.20 (0.12-0.28)	0.08 (-0.01-0.18)	0.04 (-0.07-0.14)	0.07 (-0.12-0.25)
40-<60y	0.23 (0.14-0.32)	0.14 (-0.05-0.33)	0.29 (-0.08-0.65)	0.09 (-0.32-0.50)
≥60y	0.39 (0.23-0.54)	0.37 (-0.11-0.85)	0.48 (-0.45-1.42)	

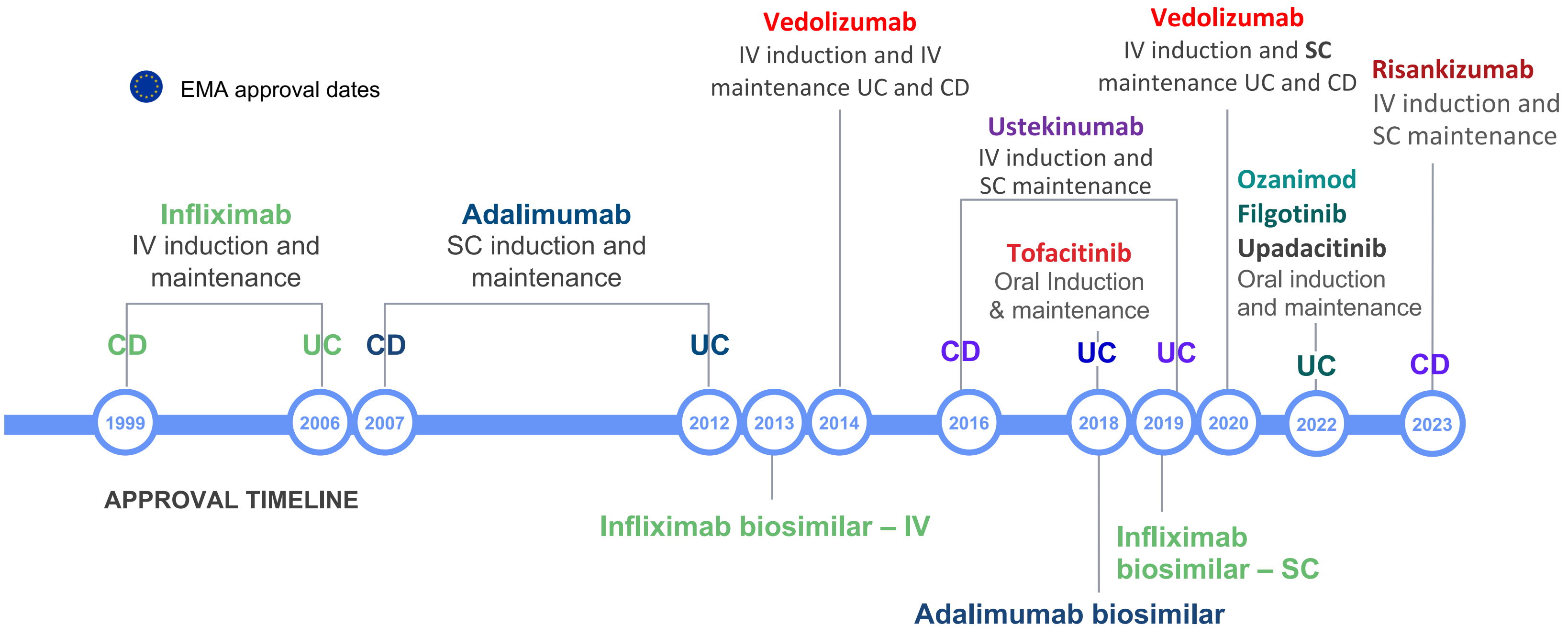

Figure S1. Overview of timeline for approval of biologic drugs in Sweden

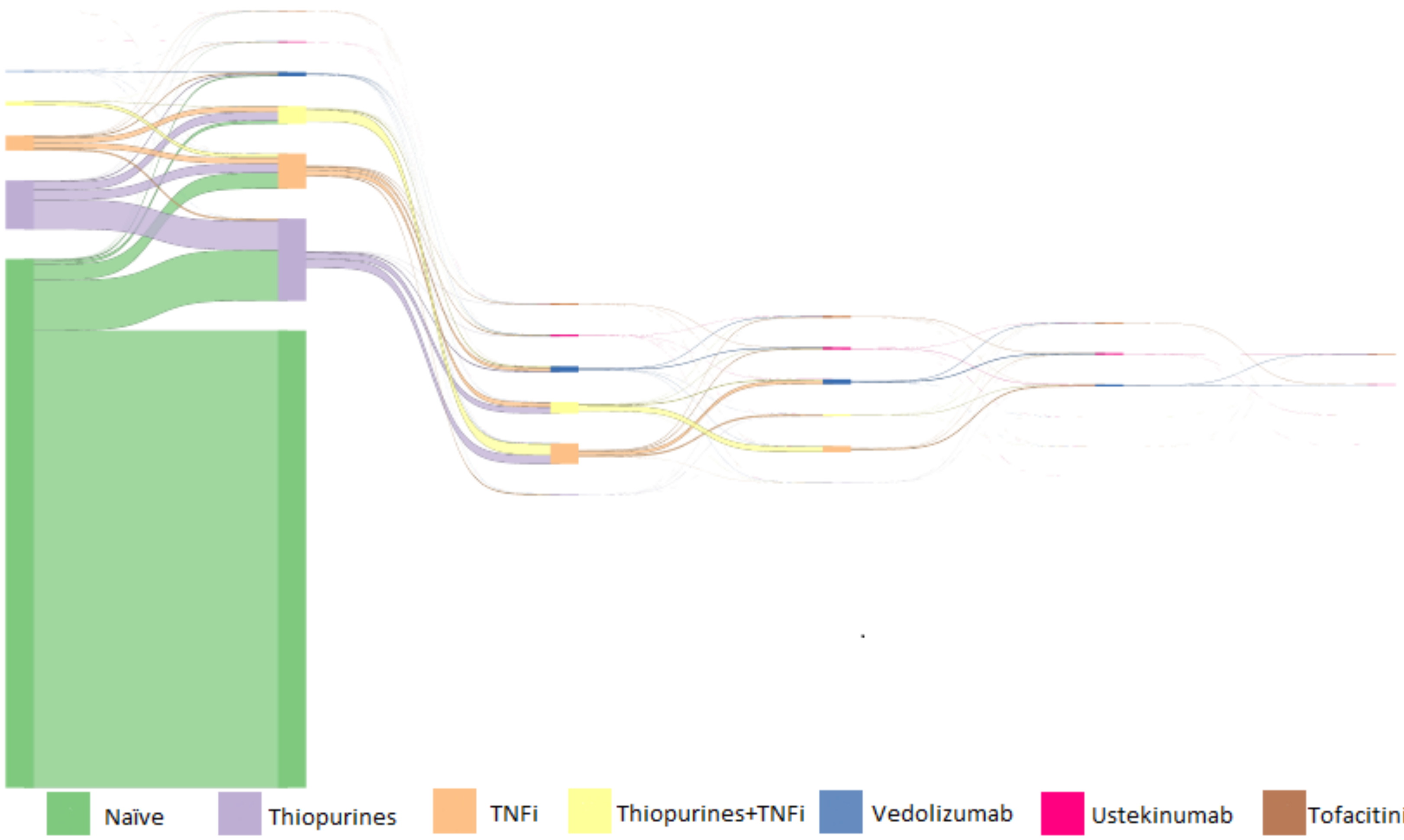

Figure S2. Number of patients remaining within the same treatment cohort or starting follow-up in another treatment cohort:
Green: cohort of patients naïve to thiopurines, tumor necrosis factor inhibitors (TNFi) and other targeted therapies at start of follow-up, Purple: Cohort of patients treated with thiopurine at start of follow-up, Orange: Cohort of patients treated with TNFi at start of follow-up, Yellow:

Cohort of patients treated with TNFi+thiopurine at start of follow-up, Blue: Cohort of patients treated with Vedolizumab at start of follow-up, Red: Cohort of patients treated with Ustekinumab at start of follow-up, Brown: Cohort of patients treated with Tofaniticib at start of follow-up

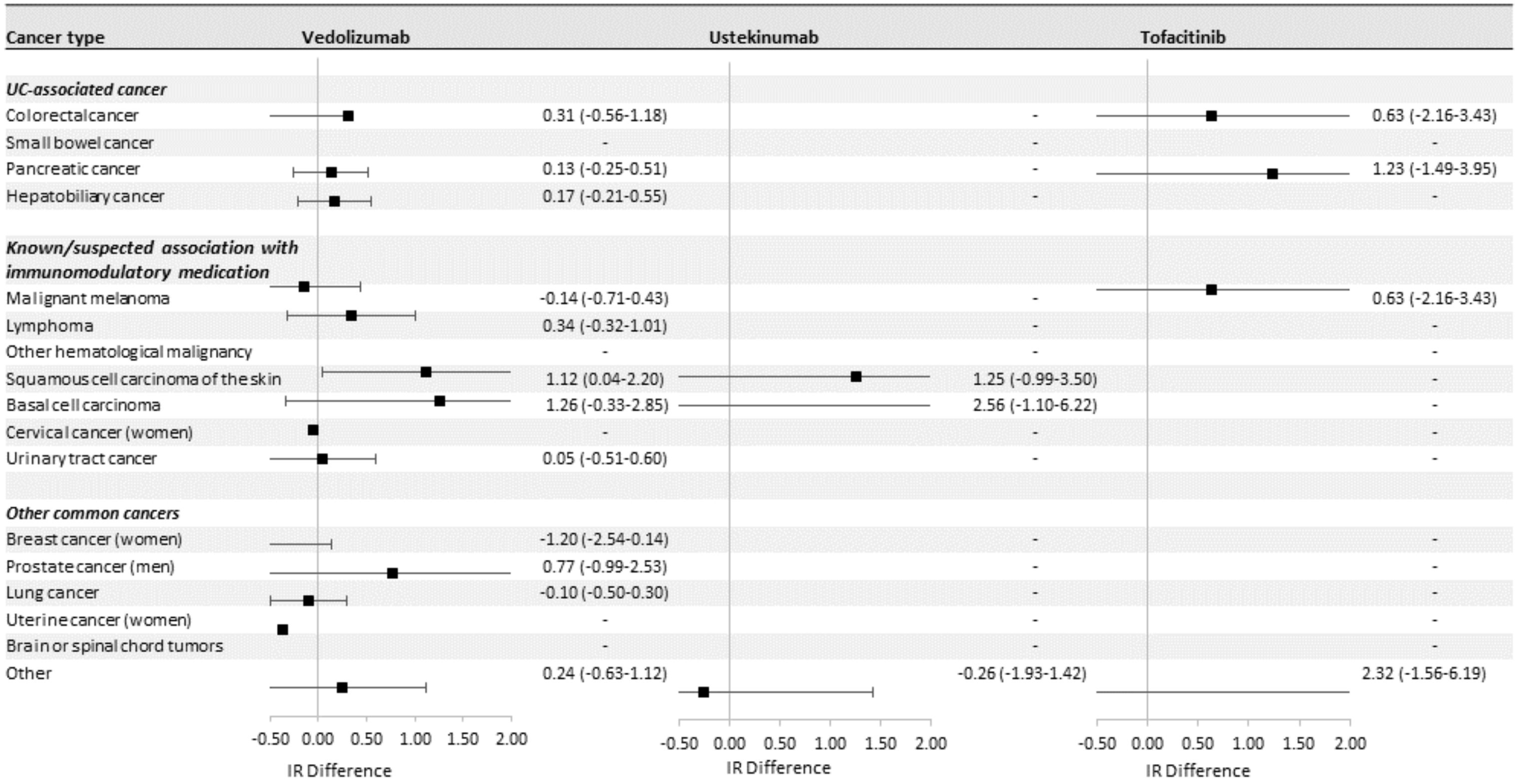

Figure S3. Incidence rate (IR) differences (cases/1000 person years) and hazard ratios with 95% confidence intervals (Cis) of of UC-associated cancers, cancers with known/suspected association with thiopurine treatment, and cancers common in the population in cohorts of patients with ulcerative colitis versus matched general population comparators, stratified by treatment at start of follow-up: vedolizumab, ustekinumab, and tofacitinib

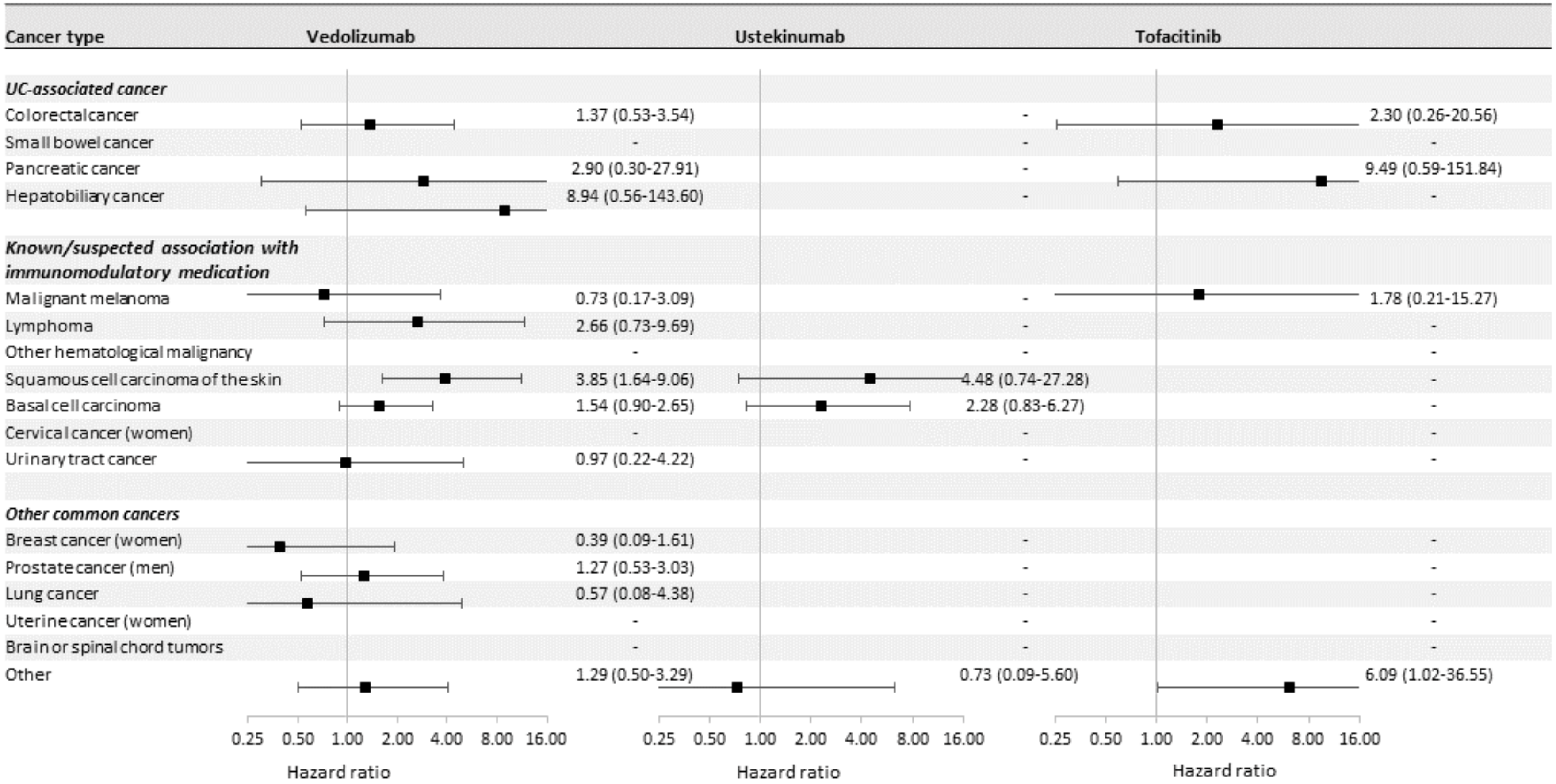

Figure S4. Hazard ratios with 95% confidence intervals (Cis) of of UC-associated cancers, cancers with known/suspected association with immunomodulatory treatment, and cancers common in the population in cohorts of patients with ulcerative colitis versus matched general population comparators, stratified by treatment at start of follow-up: vedolizumab, ustekinumab, and tofacitinib

References

1. Williams CJ, Peyrin-Biroulet L, Ford AC. Systematic review with meta-analysis: malignancies with anti-tumour necrosis factor-alpha therapy in inflammatory bowel disease. Alimentary pharmacology & therapeutics. 2014;39(5):447-58.
2. Bonovas S, Fiorino G, Allocca M, Lytras T, Nikolopoulos GK, Peyrin-Biroulet L, et al. Biologic Therapies and Risk of Infection and Malignancy in Patients With Inflammatory Bowel Disease: A Systematic Review and Network Meta-analysis. Clinical gastroenterology and hepatology : the official clinical practice journal of the American Gastroenterological Association. 2016;14(10):1385-97 e10.
3. Bezzio C, Vernero MA-O, Ribaldone DA-O, Alimenti E, Manes G, Saibeni SA-O. Cancer Risk in Patients Treated with the JAK Inhibitor Tofacitinib: Systematic Review and Meta-Analysis. LID - 10.3390/cancers15082197 [doi] LID - 2197. (2072-6694 (Print)).
4. Russell MD, Stovin C, Alveyn E, Adeyemi O, Chan CKD, Patel V, et al. JAK inhibitors and the risk of malignancy: a meta-analysis across disease indications. Annals of the rheumatic diseases. 2023;82(8):1059-67.
5. Piovani DA-O, Danese SA-O, Peyrin-Biroulet LA-O, Nikolopoulos GA-O, Bonovas SA-O. Systematic review with meta-analysis: biologics and risk of infection or cancer in elderly patients with inflammatory bowel disease. (1365-2036 (Electronic)).
6. Borren NZ, Ananthakrishnan AN. Safety of Biologic Therapy in Older Patients With Immune-Mediated Diseases: A Systematic Review and Meta-analysis. (1542-7714 (Electronic)).
7. Elmahdi R, Lemser CE, Thomsen SB, Allin KH, Agrawal M, Jess T. Development of Cancer Among Patients With Pediatric-Onset Inflammatory Bowel Disease: A Meta-analysis of Population-Based Studies. (2574-3805 (Electronic)).
8. Zhu Z, Mei Z, Guo Y, Wang G, Wu T, Cui X, et al. Reduced Risk of Inflammatory Bowel Disease-associated Colorectal Neoplasia with Use of Thiopurines: a Systematic Review and Meta-analysis. Journal of Crohn's & colitis. 2018;12(5):546-58.
9. Lu MJ, Qiu XY, Mao XQ, Li XT, Zhang HJ. Systematic review with meta-analysis: thiopurines decrease the risk of colorectal neoplasia in patients with inflammatory bowel disease. Alimentary pharmacology & therapeutics. 2018;47(3):318-31.
10. Jess T, Lopez A, Andersson M, Beaugerie L, Peyrin-Biroulet L. Thiopurines and risk of colorectal neoplasia in patients with inflammatory bowel disease: a meta-analysis. (1542-7714 (Electronic)).
11. Wijnands AM, de Jong ME, Lutgens M, Hoentjen F, Elias SG, Oldenburg B, et al. Prognostic Factors for Advanced Colorectal Neoplasia in Inflammatory Bowel Disease: Systematic Review and Meta-analysis. Gastroenterology. 2021;160(5):1584-98.
12. Chupin A, Perduca V, Meyer A, Bellanger C, Carbonnel F, Dong C. Systematic review with meta-analysis: comparative risk of lymphoma with anti-tumour necrosis factor agents and/or thiopurines in patients with inflammatory bowel disease. Alimentary pharmacology & therapeutics. 2020;52(8):1289-97.
13. Yang C, Huang J, Huang X, Huang S, Cheng J, Liao W, et al. Risk of Lymphoma in Patients With Inflammatory Bowel Disease Treated With Anti-tumour Necrosis Factor Alpha Agents: A Systematic Review and Meta-analysis. (1876-4479 (Electronic)).
14. Kotlyar DS, Lewis JD, Beaugerie L, Tierney A, Brensinger CM, Gisbert JP, et al. Risk of lymphoma in patients with inflammatory bowel disease treated with azathioprine and 6-mercaptopurine: a meta-analysis. (1542-7714 (Electronic)).

15. Singh S, Nagpal SJ, Murad MH, Yadav S, Kane SV, Pardi DS, et al. Inflammatory bowel disease is associated with an increased risk of melanoma: a systematic review and meta-analysis. (1542-7714 (Electronic)).
16. Esse S, Mason KJ, Green AC, Warren RB. Melanoma Risk in Patients Treated With Biologic Therapy for Common Inflammatory Diseases: A Systematic Review and Meta-analysis. (2168-6084 (Electronic)).
17. Huang SZ, Liu ZC, Liao WX, Wei JX, Huang XW, Yang C, et al. Risk of skin cancers in thiopurines-treated and thiopurines-untreated patients with inflammatory bowel disease: A systematic review and meta-analysis. Journal of gastroenterology and hepatology. 2019;34(3):507-16.
18. Ariyaratnam J, Subramanian V. Association between thiopurine use and nonmelanoma skin cancers in patients with inflammatory bowel disease: a meta-analysis. (1572-0241 (Electronic)).
19. Kim J, Jung JH, Jo H, Kim MH, Kang DR, Kim HM. Risk of uterine cervical cancer in inflammatory bowel disease: a systematic review and meta-analysis. (1502-7708 (Electronic)).
20. Mann SA-O, Jess T, Allin K, Elmahdi R. Risk of Cervical Cancer in Inflammatory Bowel Disease: A Meta-Analysis of Population-Based Studies. (2155-384X (Electronic)).
21. Yu JA-O, Refsum EA-O, Wieszczy P, Helsingen LM, Perrin V, Högdén A, et al. Risk of malignant lymphomas in patients with inflammatory bowel disease: a population-based cohort study. LID - 10.1136/bmjgast-2022-001037 [doi] LID - e001037. (2054-4774 (Print)).
22. Charkaoui M, Hajage D, Tubach F, Beaugerie L, Kirchgesner J. Impact of Anti-tumour Necrosis Factor Agents on the Risk of Colorectal Cancer in Patients with Ulcerative Colitis: Nationwide French Cohort Study. (1876-4479 (Electronic)).
23. Rezazadeh Ardabili AA-OX, Jeuring S, Mujagic Z, Oostenbrug L, Romberg-Camps M, Jonkers D, et al. Classic drugs in the time of new drugs: real-world, long-term outcomes of thiopurine monotherapy in 1016 patients with inflammatory bowel disease. (1365-2036 (Electronic)).
24. Ludvigsson JF, Almqvist C, Bonamy AK, Ljung R, Michaelsson K, Neovius M, et al. Registers of the Swedish total population and their use in medical research. European journal of epidemiology. 2016;31(2):125-36.
25. Ludvigsson JF, Svedberg P, Olen O, Bruze G, Neovius M. The longitudinal integrated database for health insurance and labour market studies (LISA) and its use in medical research. European journal of epidemiology. 2019;34(4):423-37.
26. Ludvigsson JF, Andersson E, Ekbom A, Feychting M, Kim JL, Reuterwall C, et al. External review and validation of the Swedish national inpatient register. BMC public health. 2011;11:450.
27. Ludvigsson JF, Andersson M, Bengtsson J, Eberhardson M, Fagerberg UL, Grip O, et al. Swedish Inflammatory Bowel Disease Register (SWIBREG) - a nationwide quality register. Scandinavian journal of gastroenterology. 2019;54(9):1089-101.
28. Wettermark B, Hammar N, Fored CM, Leimanis A, Otterblad Olausson P, Bergman U, et al. The new Swedish Prescribed Drug Register--opportunities for pharmacoepidemiological research and experience from the first six months. Pharmacoepidemiology and drug safety. 2007;16(7):726-35.
29. Socialstyrelsen. Kodning i Cancerregistret 2022 2022 [Available from: https://www.socialstyrelsen.se/globalassets/sharepoint-dokument/artikelkatalog/ovrigt/2022-3-7793.pdf.
